# 40 Hz light flickering promotes sleep through cortical adenosine signaling

**DOI:** 10.1038/s41422-023-00920-1

**Published:** 2024-02-08

**Authors:** Xuzhao Zhou, Yan He, Tao Xu, Zhaofa Wu, Wei Guo, Xi Xu, Yuntao Liu, Yi Zhang, Huiping Shang, Libin Huang, Zhimo Yao, Zewen Li, Lingya Su, Zhihui Li, Tao Feng, Shaomin Zhang, Olivia Monteiro, Rodrigo A. Cunha, Zhi-Li Huang, Kang Zhang, Yulong Li, Xiaohong Cai, Jia Qu, Jiang-Fan Chen

**Affiliations:** 1 The Eye and Brain Center, State Key Laboratory of Ophthalmology, Optometry and Visual Science, Eye Hospital, Wenzhou Medical University, Wenzhou, Zhejiang China; 2 Oujiang Laboratory (Zhejiang Laboratory for Regenerative Medicine, Vision and Brain Health), School of Ophthalmology & Optometry and Eye Hospital, Wenzhou Medical University, Wenzhou, Zhejiang China; 3 State Key Laboratory of Membrane Biology, School of Life Sciences, Peking University, Beijing, China; 4 Department of Pediatrics, The Second Affiliated Hospital and Yuying Children’s Hospital of Wenzhou Medical University, Wenzhou, Zhejiang China; 5 Institute of Genomic Medicine, Wenzhou Medical University, Wenzhou, Zhejiang China; 6 Key Laboratory of Biomedical Engineering of Ministry of Education, Qiushi Academy for Advanced Studies, Zhejiang University, Hangzhou, Zhejiang China; 7 Faculty of Medicine, Macau University of Science and Technology, Taipa, Macau China; 8 CNC-Center for Neuroscience and Cell Biology, Faculty of Medicine, University of Coimbra, Coimbra, Portugal; 9 Department of Pharmacology, School of Basic Medical Sciences; State Key Laboratory of Medical Neurobiology and MOE Frontiers Center for Brain Science, and Institutes of Brain Science, Fudan University, Shanghai, China

**Keywords:** Biological techniques, Molecular biology

## Abstract

Flickering light stimulation has emerged as a promising non-invasive neuromodulation strategy to alleviate neuropsychiatric disorders. However, the lack of a neurochemical underpinning has hampered its therapeutic development. Here, we demonstrate that light flickering triggered an immediate and sustained increase (up to 3 h after flickering) in extracellular adenosine levels in the primary visual cortex (V1) and other brain regions, as a function of light frequency and intensity, with maximal effects observed at 40 Hz frequency and 4000 lux. We uncovered cortical (glutamatergic and GABAergic) neurons, rather than astrocytes, as the cellular source, the intracellular adenosine generation from AMPK-associated energy metabolism pathways (but not SAM-transmethylation or salvage purine pathways), and adenosine efflux mediated by equilibrative nucleoside transporter-2 (ENT2) as the molecular pathway responsible for extracellular adenosine generation. Importantly, 40 Hz (but not 20 and 80 Hz) light flickering for 30 min enhanced non-rapid eye movement (non-REM) and REM sleep for 2–3 h in mice. This somnogenic effect was abolished by ablation of V1 (but not superior colliculus) neurons and by genetic deletion of the gene encoding ENT2 (but not ENT1), but recaptured by chemogenetic inhibition of V1 neurons and by focal infusion of adenosine into V1 in a dose-dependent manner. Lastly, 40 Hz light flickering for 30 min also promoted sleep in children with insomnia by decreasing sleep onset latency, increasing total sleep time, and reducing waking after sleep onset. Collectively, our findings establish the ENT2-mediated adenosine signaling in V1 as the neurochemical basis for 40 Hz flickering-induced sleep and unravel a novel and non-invasive treatment for insomnia, a condition that affects 20% of the world population.

## Introduction

One of the most significant environmental factors affecting human physiology is light. As a non-invasive neuromodulation strategy, light stimulation has demonstrated potential for alleviating various pathological changes in animal models of depression,^1,2^ insomnia,^3^ and Alzheimer’s disease (AD).^4^ In particular, light flickering at 40 Hz is receiving increased attention for its ability to reverse pathological features of AD,^4–6^ ischemia,^7^ and traumatic brain injury^8^ in animals. The demonstration of gamma oscillation entrainment across multiple brain regions, including the hippocampus,^5^ and the reduction of amyloid loading with activated microglia and increased vascular densities and improvement of cognitive performance by combined visual and acoustic stimulation (GENUS) in AD mice,^4,6^ has led to the US FDA designating the 40 Hz flicker paradigm (GENUS equipment) as a “breakthrough device” in 2021 for further clinical investigation.^9^ Light flickering-induced neurochemical coupling mechanisms are the key to its therapeutic effect. Current research focuses on neural activity and oscillation induced by light, such as gamma oscillation entrainment,^5,6,10^ which is hypothesized to be a prerequisite, but not sufficient to account for the therapeutic effect. The neurochemical and molecular adaptations are critical determinants for the biological effects of 40 Hz light flickering as the treatment often requires more than 2 weeks to 2 months to achieve effective therapeutic benefits.^4–6^ 40 Hz flickering reportedly alters neuroimmune signaling^11,12^ and neuromodulators such as acetylcholine,^10^ and gene expression profiles of many brain cell types^5^ were elevated following exposure. Yet, specific neurochemical basis for 40 Hz flickering effects remains undetermined, contributing to questions on variable effects of 40 Hz flickering.^10^ Thus, it is paramount to delineate the neurochemical mechanisms underlying 40 Hz light flickering to fully exploit its therapeutic potential.

Light flickering-triggered neural activity (e.g., spike activity or gamma oscillation) is especially energy demanding,^12^ requiring robust mitochondrial function and producing strong oxygen consumption with hemodynamic changes, as evidenced by blood oxygen level-dependent signals in the cortex.^13,14^ Given that adenosine is the main signaling molecule resulting from increased energy expenditure,^15–17^ we reasoned that light flickering-triggered neural activity represents a form of energy-demanding neural stimulation that leads to rapid adenosine generation. Extracellular adenosine, with its intrinsic link with energy metabolism, can simultaneously act as a modulator of neurotransmission and synaptic plasticity,^18^ and a homeostasis regulator of metabolism, motility, proliferation, and vasodilation.^16,19^ Therefore, we hypothesized that adenosine is crucial to the therapeutic effect observed with 40 Hz light flickering.

Among its multiple physiological and pathophysiological effects, adenosine is a well-known physiological regulator of homeostatic sleep requirements. Extracellular adenosine levels in the basal forebrain rise after prolonged wakefulness, resulting in increased sleep pressure.^20–22^ Adenosine’s somnogenic effect can be highlighted through the intracerebral or systemic administration of adenosine and its analogs or by pharmacological modulation of adenosine metabolism.^23^ Conversely, caffeine, the most widely consumed psychostimulant and a non-selective adenosine receptor antagonist, produces a strong arousal effect.^22–24^ Based on this reasoning, we propose that 40 Hz light flickering may be an effective somnogen by inducing an increase in cortical adenosine levels. The adenosine hypothesis of 40 Hz flickering may not only provide a neurochemical explanation for some observations that 40 Hz flickering produces drowsiness in AD patients^9^ and improves β-amyloid-induced sleep disturbance^25^ and rhythm disorder,^26^ but also offer a novel and non-invasive therapy for chronic insomnia, which affects 20% of the world population^27^ and up to 76% of these people with comorbidities.^28^

In this study, we established that cortical adenosine signaling is crucial for the biological effects of 40 Hz light flickering on sleep. We demonstrated here that light flickering triggered a rapid and sustained increase in extracellular adenosine levels in the visual cortex and other brain regions, which depended on the light flickering frequency, intensity, and wavelength. We identified cortical (glutamatergic and GABAergic) neurons as the cellular source and the intracellular adenosine generation from energy metabolism and adenosine efflux mediated by equilibrative nucleoside transporter-2 (ENT2, but not ENT1) as the molecular cascade responsible for extracellular adenosine generation by 40 Hz flickering. Furthermore, we uncovered that 40 Hz flickering induced somnogenic effect by modulating primary visual cortical (V1) activity via adenosine. Lastly, 40 Hz light flickering for 30 min promoted sleep onset and maintenance in mice and children with insomnia. Collectively, we have defined ENT2-mediated adenosine signaling in V1 as the neurochemical basis for 40 Hz flickering-induced sleep, and developed a novel and non-invasive treatment for insomnia with potentially wider therapeutic implications.

## Results

### 40 Hz light flickering increases extracellular adenosine levels in frequency- and intensity-dependent manners in the visual cortex and other brain regions

**Fig. 1:**
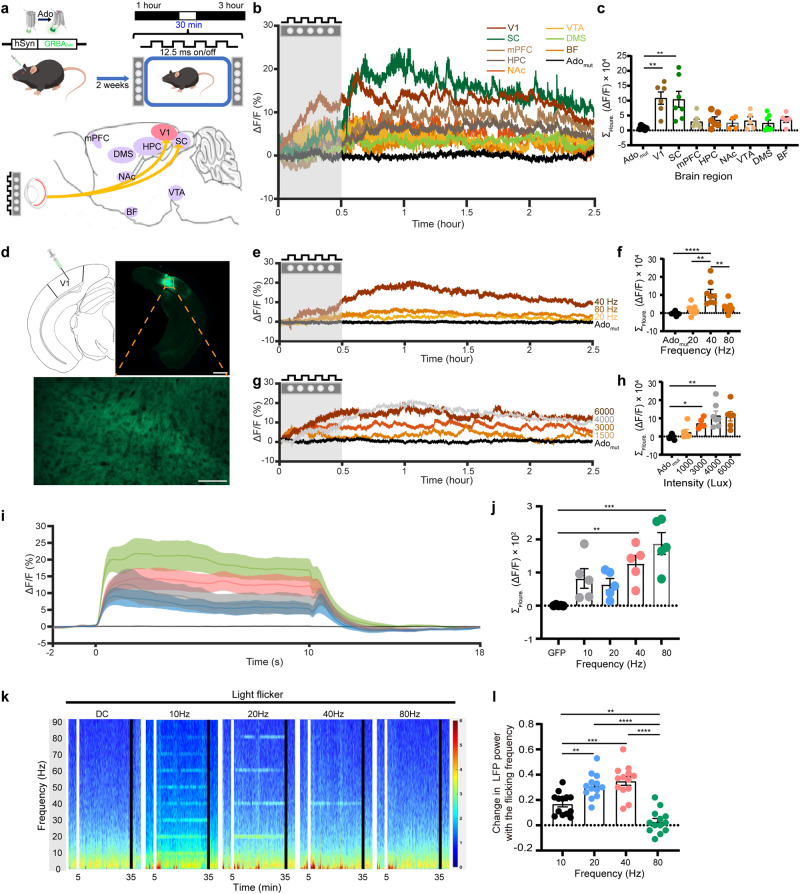
40 Hz light flickering increases extracellular adenosine levels in light frequency- and intensity-dependent manners in the primary visual cortex in correlation with LFP gamma power. **a** Schematic diagram depicting the experimental configuration for visual stimulation (upper panel). Schematic diagram depicting fiber photometry recording of extracellular adenosine levels in eight brain regions of mice, including V1, BF, NAc, VTA, SC, mPFC, HPC, and DMS using GRAB_Ado1.0_. (lower left panel). **b** The effect of 40 Hz light flickering (4000 lux) on extracellular adenosine levels in eight brain regions of mice, including V1, BF, NAc, VTA, SC, mPFC, HPC, and DMS (*n* = 5–7/group). The vertical line in each plot represents the start and end of the light-flickering period. **c** Quantification of light flickering-evoked adenosine signals in V1, NAc, BF, VTA, SC, mPFC, HPC, and DMS (*n* = 5–7/group). **d** Fluorescence image of V1 showing the expression of hSyn-GRAB_Ado1.0_. Scale bar = 500 μm (the upper right panel); Scale bar = 300 μm (the lower panel). **e** Light flickering at 20, 40, and 80 Hz induced an immediate (within minutes) and sustained (lasting for up to 2 h) increase in the extracellular adenosine levels in V1 during and after flickering, with 40 Hz flickering yielding the maximum increase (*n* = 7/group). **f** Quantification of light flickering-evoked adenosine signals in V1 at 20, 40, and 80 Hz (*n* = 7/group). **g** The effect of 40 Hz white light flickering with different intensities (1500–6000 lux) on extracellular adenosine levels in V1 (*n* = 5/group). **h** Quantification of light flickering-evoked adenosine signals in V1 at 1500, 3000, 4000 and 6000 lux (*n* = 5–8/group). **i** Light flickering at 10, 20, 40, and 80 Hz (50% duty cycle) produced a linear rise in calcium signals of vGluT2^+^ neurons in V1 when we compared the “On time” (10 s) with the “Off time”. **j** Quantification of light flicker producing the calcium peak at 10, 20, 40, and 80 Hz (*n* = 5/group). **k** Representative time-resolved spectrograms of the primary visual cortex (V1) during 10, 20, 40, and 80 Hz light flickering and DC stimulation. The vertical line in each plot represents the start and end of the light-flickering period. **l** Quantified and normalized values of power in the flickering frequency during flickering (*n* = 13/group). The data were analyzed using repeated measure ANOVA test followed by Turkey’s comparisons test. The data are presented as mean ± SEM or upper and lower quartile (IQR) and median (boxplot), ***P < 0.001, **P < 0.01, *P < 0.05.

To examine the role of extracellular adenosine in the biological effects of 40 Hz light flickering, we utilized a highly sensitive and selective G protein-coupled receptor (GPCR)-activation-based adenosine sensor (GRAB_Ado_) to monitor changes in extracellular adenosine levels in response to 40 Hz light flickering.^21^ First, we injected an AAV expressing GRAB_Ado_ or a non-binding mutant of GRAB_Ado_ into the brains of mice at eight different brain regions that were activated (as indicated by c-Fos expression) by 40 Hz flickering,^29^ and inserted a multi-fiber probe to record the regional variations in extracellular adenosine levels induced by 40 Hz light flickering (Fig. 1a). We employed fiber photometry to assess fluorescence signals indicative of extracellular adenosine. Two weeks later after virus injections, after exposing mice to white light flickering (40 Hz, illuminance was 4000 lux or irradiance was 1.22 mW/cm^2^, 50% duty cycle) produced by a custom-made LED device^4,30^ (see below for characterization of light frequencies and intensities), we found there was a robust and sustained increase in extracellular adenosine levels in V1 and the superior colliculus (SC), with a ∆*F*/*F* of 20%–30% above the baseline. We also found a moderate increase in the basal forebrain (BF) and prefrontal cortex (mPFC) (∆*F*/*F* of 5%–10%), hippocampus (HPC), nucleus accumbens (NAc), and the ventral tegmental area (VTA) (∆*F*/*F* of 0%–5%), as depicted in Fig. 1b, c. The temporal features of adenosine elevation induced by 40 Hz flickering were similar across these brain regions, with little to no increase observed in the dorsomedial striatum (DMS) (Fig. 1b, c). In addition to the immediate surge in extracellular adenosine levels, we observed a substantial and delayed increase in adenosine levels in V1 after the cessation of 40 Hz light flickering (Fig. 1b). This increase reached its peak (with ∆*F*/*F* value up to 15%–20% higher than the baseline) at 30 min and lasted for up to 3 h before returning to the baseline levels (Fig. 1b). The specificity of this extracellular adenosine generation was validated by the absence of fluorescent signal increases in V1 neurons expressing the non-binding mutant of GRAB_Ado_ (Fig. 1b, black line).

Since V1 is likely the entrance route for the 40 Hz-induced biological effect with robust adenosine elevations, we further characterized the impact of different light frequencies (20, 40 and 80 Hz) and varying intensities of white light (1500, 3000, 4000, and 6000 lux) on extracellular adenosine production in V1 in response to light flickering (Fig. 1d). We found that the effect of light flickering on extracellular adenosine levels in the visual cortex was frequency-dependent: 40 Hz light flickering produced the maximum increase in extracellular adenosine levels that lasted the longest (2–3 h), while light flickering at 20 or 80 Hz resulted in discrete changes in extracellular adenosine levels in V1 (Fig. 1e, f). Thus, the frequency of light flickering plays a crucial role in determining the increase in extracellular adenosine levels in V1.

Moreover, we characterized the impact of varying intensities of white light (1500, 3000, 4000, and 6000 lux) on extracellular adenosine production and found that 40 Hz flickering at high light intensities (4000 and 6000 lux) induced robust (with a peak ∆*F*/*F* ~ 20%) and sustained (up to 3 h) increase of extracellular adenosine in V1 (Fig. 1g). By contrast, 40 Hz flickering at low intensities (<1500 lux) produced relatively moderate increase of extracellular adenosine with shorter duration (Fig. 1g, h). Thus, we have used 40 Hz frequency and 4000 lux (i.e., 1.22 mW/cm^2^ at 20 cm distance) for the rest of the experiment to analyze the impact of light flickering at the cellular and molecular levels. It should be noted that brief (30 min) light intensity is well tolerated within the safety limit in mice (5000 lux light within 10 h).^2,31,32^ Indeed, 40 Hz light flickering with 4000 lux (1.22 mW/cm^2^ at 20 cm distance, 30 min/day) for 30 min daily and continued for 14 days in mice did not affect the intraocular pressure (IOP) and retinal thickness (Supplementary information, Fig. S1g–i).

We sought to establish an electrophysiological correlation between extracellular adenosine generation and neural activity (as indicated by calcium signal) or gamma oscillation power (as detected by local field potential (LFP)) in response to light flickering at different frequencies before, during, and after the light flickering phase (30 min). Fiber optometry recording revealed a linear increase in calcium signals in V1 with increasing frequency. Specifically, light flickering at 10, 20, 40, and 80 Hz resulted in a linear rise in calcium signals in V1, with the strongest response observed at 80 Hz, producing the highest amplitude of the calcium peak (Fig. 1i, j). Furthermore, we found that felodipine (10 mg/kg, intraperitoneal injection), a VGCC blocker, almost completely abolished the 40 Hz flickering-induced increase in extracellular adenosine levels compared to the vehicle group (Supplementary information, Fig. S2a, b). These results suggest that while neural activity (as indicated by calcium signal) is required for the induction of adenosine release, it does not correlate with the frequency specificity of light flickering. However, we found that the LFP oscillation was frequency-dependent, where each frequency of light flickering elicited specific LFP oscillation (Fig. 1k, l). Specifically, light flickering at 10, 20, and 40 Hz induced LFP oscillations distinctively at the alpha, theta, and gamma ranges, respectively. However, 80 Hz light flickering and direct current (DC) failed to induce oscillations at the gamma band (Fig. 1k, l). When comparing the LFP power in the flickering frequency, we found that the normalized power significantly increased at both 20 and 40 Hz compared to 10 Hz (40 Hz vs 10 Hz, *P* < 0.0001; 20 Hz vs 10 Hz, *P* = 0.0052) (Fig. 1l). Since gamma oscillation has been shown to be strongly correlated with a hemodynamic response (i.e., energy demand),^13^ and only 40 Hz light flickering induced LFP power increase in the gamma frequency, 40 Hz light flickering had the highest induced energy expenditure compared to the other frequencies.

### Glutamatergic and GABAergic neurons are the main cellular source of light flickering-induced adenosine generation in V1

**Fig. 2:**
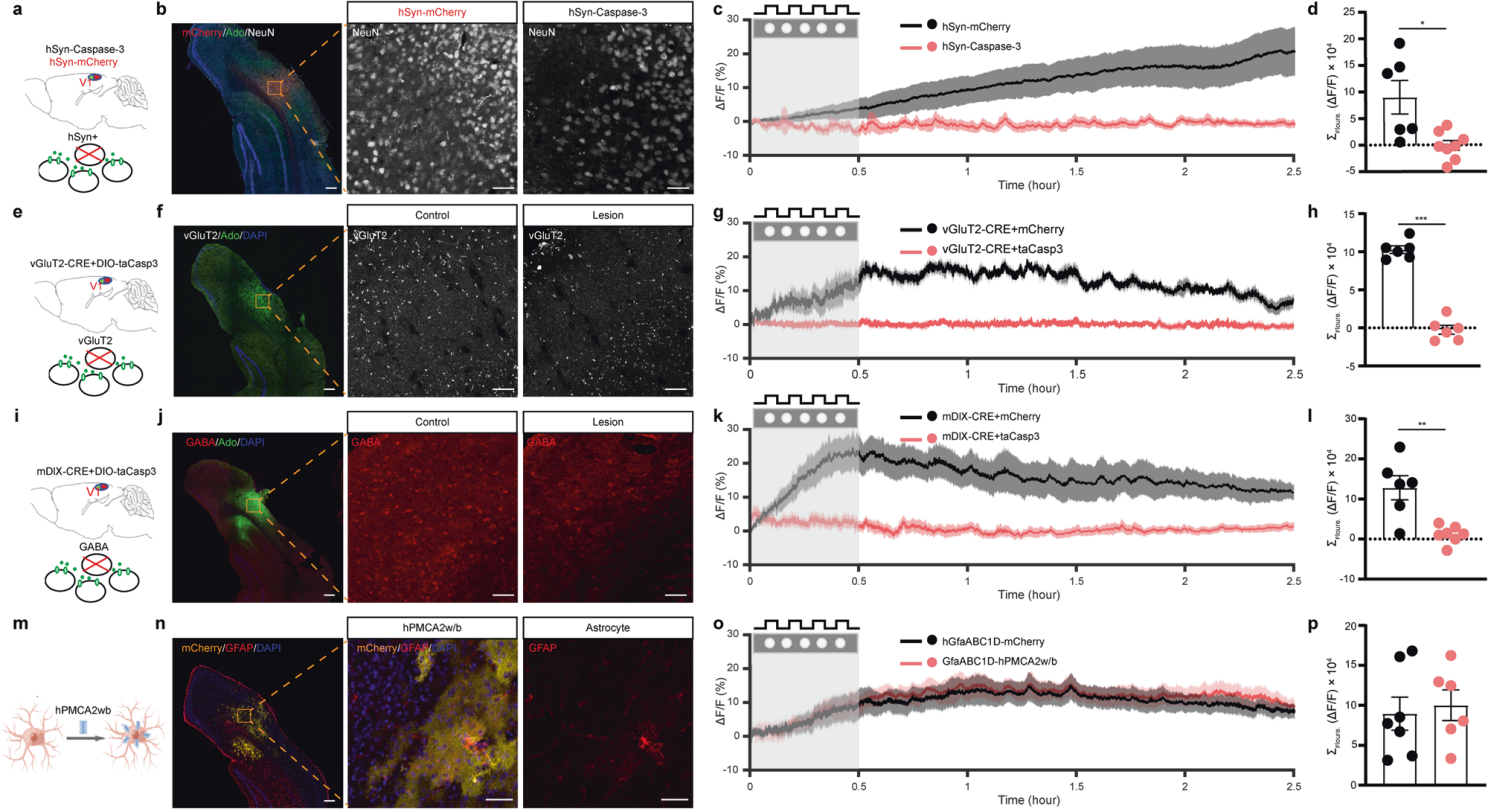
Glutamatergic and GABAergic neurons are the primary cellular source for 40 Hz flickering-induced extracellular adenosine generation in V1. **a** Schematic diagram depicting the fiber photometry recording of extracellular adenosine levels after selective ablation of total neurons in V1 using Caspase3. **b** Fluorescence image of V1 showing the staining of NeuN in V1 carrying AAV2/9-EF1a-flex-taCaspase3 or AAV2/9-EF1a-flex-mCherry. **c** Ablation of V1 neurons significantly reduced the generation of extracellular adenosine in response to 40 Hz light flickering compared to the control side. **d** Quantification of 40 Hz light flickering-evoked adenosine signals with Caspase3-ablated neurons in V1 compared to control mice (*n* = 6–7/group). **e** Schematic diagram depicting the strategy used to selectively ablate vGluT2^+^ neurons in the visual cortex using vGluT2-CRE mice coupled with AAV2/9-EF1a-flex-taCaspase3. **f** Fluorescence image of V1 showing the reduction of vGluT2^+^ neurons in V1 expressing AAV2/9-EF1a-flex-taCaspase3 compared to the cortex expressing AAV2/9-EF1a-flex-mCherry (lower panel). **g** Ablation of vGluT2^+^ neurons in V1 suppressed the generation of extracellular adenosine in response to 40 Hz flickering compared to control mice. **h** Quantification of 40 Hz light flickering-evoked adenosine signals between ablated and non-ablated vGluT2^+^ neurons in V1 (*n* = 6/group). **i** Schematic diagram depicting the strategy used to selectively ablate GABA neurons in V1 using AAV2/9-mDIX-CRE coupled with AAV2/9-EF1a-flex-taCaspase3. **j** Fluorescence image of V1 showing the reduction of GAD^+^ neurons in V1 expressing AAV2/9-EF1a-flex-taCaspase3 compared to V1 expressing AAV2/9-EF1a-flex-mCherry. **k** Ablation of the GAD^+^ neurons in V1 suppressed the generation of extracellular adenosine in response to 40 Hz flickering compared to control mice. **l** Quantification of 40 Hz light flickering-evoked adenosine signals between ablated and non-ablated GAD^+^ neurons in V1 (*n* = 6–7/group). **m** Expression of hPMCA2w/b in V1 partially reduced astrocytic activity. **n** Immunohistochemical images showing the co-localization of GFAP and hPMCA2w/b in V1 astrocytes. **o** Inhibition of astrocytic activity in V1 produced a comparable generation of extracellular adenosine in response to 40 Hz flickering compared to the control side. **p** Partial inhibition of astrocytic activity by hPMCA2w/b expression did not affect extracellular adenosine generation in response to 40 Hz flickering (*n* = 6–7/group). ***P < 0.001, **P < 0.01, *P < 0.05, Student’s *t*-test. The data are mean ± SEM. Scale bar = 100 μm in **b**, **f**, **j**, **n**.

As adenosine can be released from both astrocytes and neurons,^17,33^ we investigated the cellular source responsible for the extracellular adenosine generation induced by 40 Hz light flickering in V1. First, we investigated the role of cortical neurons in extracellular adenosine generation induced by 40 Hz light flickering using AAV2/9-hSyn-taCaspase3-T2A-TEVp to induce selective neuronal apoptosis.^21^ We co-injected AAVs carrying glial-specific GfaABC1D-GRAB_Ado_ and neuron-specific hSyn-taCaspase3 into the visual cortex of one hemisphere and GfaABC1D-GRAB_Ado_ with hSyn-mCherry into the other hemisphere (Fig. 2a). Two weeks after achieving targeted expression, we confirmed the selective reduction in the number of neurons in V1 expressing hSyn-Caspase3 compared to the cortex expressing hSyn-mCherry using NeuN immunoreactivity (Fig. 2b). We found that in the hemisphere expressing mCherry, the extracellular adenosine levels gradually and progressively increased during 40 Hz light flickering and persisted for 150 min after the cessation of stimulation (Fig. 2c, d). Notably, the pattern of increased extracellular adenosine levels observed via GRAB_Ado_ expressed in astrocytes exhibited some dissimilarity compared to the pattern detected by GRAB_Ado_ expressed in neurons, indicating a subtle variation in local adenosine levels between neurons and astrocytes. Importantly, the visual cortex that expressed hSyn-Caspase3 did not exhibit any progressive rise in extracellular adenosine levels in response to 40 Hz light flickering. This suggests that V1 neurons are the primary cellular source of extracellular adenosine generation induced by 40 Hz light flickering.

We further dissected the specific contribution of glutamatergic neurons to extracellular adenosine generation by ablating glutamatergic neurons in the visual cortex. In vGluT2-CRE mice, we co-injected AAV2/9-EF1a-flex-taCaspase3 together with AAV2/9-hSyn-GRAB_Ado_ into one hemisphere (the visual cortex) and AAV2/9-EF1a-flex-mCherry together with AAV2/9-hSyn-GRAB_Ado_ into the other hemisphere (Fig. 2e). Two weeks after injection, we confirmed by vGluT2 immunofluorescence staining the selective reduction of vGluT2^+^ neurons in V1 expressing hSyn-Caspase3 compared to the cortex expressing hSyn-mCherry (Fig. 2f). In response to 40 Hz light flickering, V1 neurons expressing vGluT2-mCherry exhibited a substantial increase in extracellular adenosine levels (Fig. 2g, h). However, the ablation of glutamatergic neurons in V1 almost completely abolished the production of extracellular adenosine triggered by 40 Hz flickering, both during the initial and delayed phases, supporting the idea that glutamatergic neurons are an important cellular source of extracellular adenosine in response to 40 Hz light flickering.

We also determined the specific contribution of GABAergic neurons to extracellular adenosine generation by ablating GABAergic neurons in V1 via co-injecting AAV2/9-EF1a-flex-taCaspase3 and AAV2/9-mDIX-CRE together with hSyn-GRAB_Ado_ into one hemisphere and AAV2/9-EF1a-flex-mCherry and AAV2/9-mDIX-CRE together with hSyn-GRAB_Ado_ into the other hemisphere (Fig. 2i). Two weeks after the injections, we verified by GABA immunofluorescence staining the selective reduction of GABA neurons in the visual cortex expressing hSyn-Caspase3 compared to the cortex expressing hSyn-mCherry (Fig. 2j). We found that the hemisphere expressing AAV2/9-EF1a-flex-mCherry showed a robust and progressive elevation in extracellular adenosine upon exposure to 40 Hz flickering and lasted for 2.5 h after flickering cessation. In contrast, 40 Hz flickering-induced extracellular adenosine generation was largely attenuated in V1 expressing AAV2/9-EF1a-flex-taCaspase3 (Fig. 2k, l). Thus, GABAergic neurons are also a cellular source of extracellular adenosine in the visual cortex in response to 40 Hz light flickering. Gamma oscillation is conceptualized as an ability of excitatory neurons to generate spiking, which triggers synchronized activity of interconnected interneurons that feedback inhibit excitatory neurons to generate a transient silent period.^34^ When this inhibition declines, excitatory neurons spike again, leading to a new cycle of gamma. As both inhibitory and excitatory neurons are indispensable components of the local circuit network to generate gamma oscillation, lesioning of either inhibitory (GABA neurons) or excitatory (glutamatergic neurons) is expected to blunt rhythmic gamma oscillation and consequently adenosine generation. Overall, these data suggest that interactions between inhibition and excitation generate extracellular adenosine.

Lastly, we assessed the involvement of astrocytes in extracellular adenosine production induced by 40 Hz flickering by inhibiting astrocyte calcium signaling using hPMCA2w/b expression (Fig. 2m, n). We confirmed the selective expression of hPMCA2w/b in V1 astrocytes by immunohistochemistry, which showed its co-localization with GFAP immunoreactivity (Fig. 2n). We performed foot shock conditioning tasks to reveal that expression of AAV2/9-GfaABC1D-hPMCA2w/b in astrocytes partially suppressed astrocytic activation, as evidenced by a ~40% reduction in the calcium signal response in V1 compared to mice expressing AAV2/9-GfaABC1D-mCherry, which is consistent with a previous study (Supplementary information, Fig. S3a, b).^35^ We co-microinjected AAV2/9-GfaABC1D-hPMCA2w/b and GRAB_Ado_ into a single hemisphere of the visual cortex, while AAV2/9-GfaABC1D-mCherry and GRAB_Ado_ were injected into the other hemisphere. Two weeks after the injection, the increase in extracellular adenosine induced by 40 Hz flickering in V1 was not affected by the partial inhibition of astrocytic activity caused by hPMCA2w/b compared to the mCherry group (Fig. 2o, p), indicating that astrocytic activity does not play a crucial role in extracellular adenosine generation induced by light flickering. This result is consistent with a recent study showing that electric stimulation fails to induce adenosine release from astrocytes.^36^

### The ENT2-mediated efflux of intracellular adenosine is the main source for 40 Hz flickering-induced extracellular adenosine generation in V1

**Fig. 3:**
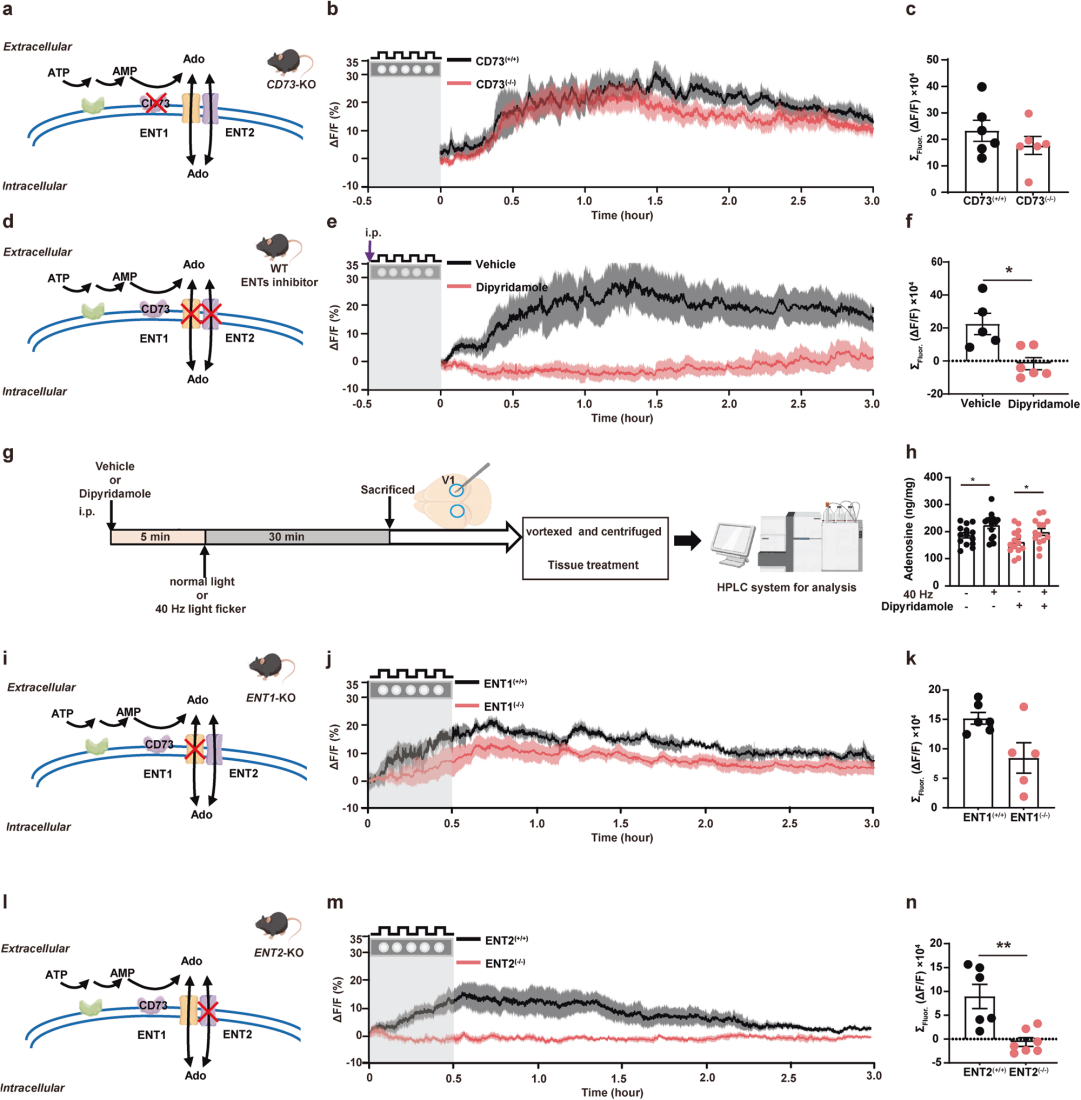
Extracellular adenosine generation in response to 40 Hz flickering in the primary visual cortex is mediated by ENT2. **a** Schematic drawing depicting the production of Adenosine (Ado) from ATP via CD73 enzymes. **b** Extracellular adenosine increase (as detected by GRAB_Ado_) caused by 40 Hz light flickering in V1 of WT and *CD73*-KO mice. **c** Quantified fluorescence of GRAB_Ado1.0_ Δ*F/F0* in response to 40 Hz light flickering in *CD73*-KO mice (*n* = 6/group). **d** Schematic drawing depicting the release of Ado via ENTs and the pharmacological inhibition of ENTs blocking activity. **e** Pretreatment with dipyridamole (30 min before light flickering) abolished 40 Hz flickering-induced extracellular adenosine generation during light flickering and after stimulation cessation. **f** Group summary of GRAB_Ado1.0_ Δ*F/F0* in response to 40 Hz light flickering application of dipyridamole (*n* = 5–6/group). **g** Description of metabolomic screening for dipyridamole administration **h** Total tissue (intracellular and extracellular) adenosine levels in V1, as assessed by HPLC analysis, after 40 Hz light flickering with or without pretreatment with dipyridamole (*n* = 14/group). *P < 0.05, comparing the dipyridamole-treated group with the vehicle-treated group. **i** Schematic drawing depicting the release of Ado via ENTs and blocking ENT1 activity in *ENT1*-KO mice. **j** 40 Hz flickering induced a robust and sustained increase of the extracellular adenosine generation in both WT and *ENT1*-KO mice, with slightly lesser induction in *ENT1*-KO than WT mice at later time points. **k** Group summary of GRAB_Ado1.0_ ΔF/F0 in response to 40 Hz light flickering from *ENT1*-KO mice (*n* = 5–6/group). **l** Schematic drawing depicting the release of Ado via ENTs and blocking ENT2 activity by *ENT2*-KO mice. **m** 40 Hz flickering-induced extracellular adenosine generation was robust and sustained in WT but largely abolished in *ENT2*-KO mice. **n** Group summary of Ado1.0 Δ*F*/*F*0 in response to 40 Hz light flickering from *ENT2*-KO mice (*n* = 6–7/group). The data are presented as mean ± SEM, **P < 0.01, *P < 0.05, Student’s *t*-test; WT vs KO; dipyridamole-treated group vs vehicle group.

We next aimed to identify the pathways responsible for the 40 Hz flickering-induced increase in extracellular adenosine that occurs after flickering stimulation cessation, distinguishing between extracellular and intracellular mechanisms. For extracellular adenosine production, 40 Hz flickering may trigger ATP release from neurons and astrocytes, followed by extracellular conversion to AMP and subsequently to adenosine via ecto-nucleotidases CD39 and CD73, respectively. We observed that extracellular adenosine levels in V1 of both wild-type (WT) and *CD73*-knockout (*CD73*-KO) mice displayed a largely similar delayed increase, peaking at 30 min and lasting for 3 h upon 40 Hz flickering stimulation (Fig. 3a–c). Thus, the extracellular formation of adenosine by ATP release (Supplementary information, Fig. S3c, d) and CD73-dependent conversion is not the primary source of the 40 Hz flickering-induced increase in extracellular adenosine in V1.

We next explored the contribution of intracellularly generated adenosine to the delayed and sustained rise of extracellular adenosine, which involves an augmented production of intracellular adenosine as a by-product of increased energy (intracellular ATP) consumption followed by an ENT1/2-mediated transmembrane efflux of adenosine. We first assessed the contribution of ENT1/2-mediated transmembrane transport of adenosine after 40 Hz flickering using the ENT inhibitor dipyridamole. We found that 40 Hz light flickering induced a robust and sustained increase (up to 2–3 h) in extracellular adenosine levels in V1 of mice treated with vehicle (0.5% methylcellulose), but this increase was almost completely abolished by pretreatment (30 min before light flickering) with dipyridamole (15 mg/kg) (Fig. 3d–f). Furthermore, when dipyridamole was administered immediately after stopping light flickering delivery, the increase in extracellular adenosine (after an initial increase during the flickering period) was largely prevented, indicating that the sustained increase in extracellular adenosine was also dependent on ENT activity (Supplementary information, Fig. S3e, f). On the other hand, 40 Hz light flickering increased the total amount of adenosine (~90% being intracellular adenosine), and the increase in the total amount of adenosine induced by 40 Hz flickering was not prevented by pretreatment with dipyridamole (Fig. 3g, h). Taken together, the observation that dipyridamole abolished the variation of extracellular adenosine levels but not the total (mainly intracellular) adenosine levels is in line with ENT-mediated adenosine efflux from the intracellular to the extracellular compartments.

The transmembrane transport of adenosine is mainly carried out by two different ENTs, with ENT1 having a relatively higher affinity and lower capacity for adenosine and ENT2 having a relatively lower affinity and higher capacity.^37^ We employed *ENT1*-KO and *ENT2*-KO mice^36^ to determine their individual contributions to 40 Hz flickering-induced extracellular adenosine generation in V1. As expected, 40 Hz flickering consistently produced a robust increase in extracellular adenosine levels in WT mice. In *ENT1*-KO mice, there was still a significant and sustained increase in extracellular adenosine levels in response to 40 Hz flashing, although slightly lower than that in WT mice at later time points (Fig. 3i–k). By contrast, the increase in extracellular adenosine generation during and after light flickering was almost completely abolished in *ENT2*-KO mice (Fig. 3l–n). These findings underscore the essential role of ENT2, rather than ENT1, in mediating the elevation of extracellular adenosine in the cortex induced by 40 Hz flickering, by promoting the transfer of adenosine from the intracellular to the extracellular compartment.

### 40 Hz flickering induces extracellular adenosine generation resulting from an AMPK-associated control of energy metabolism in V1

**Fig. 4:**
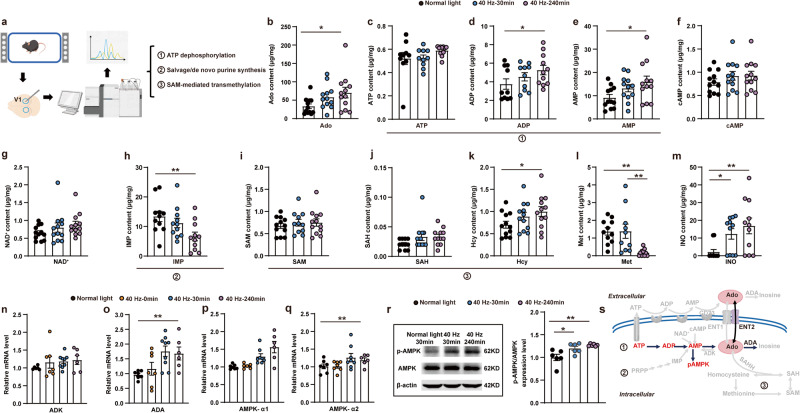
The intracellular pathway of adenosine production in response to 40 Hz flickering primarily involves energy metabolism. **a** Description of metabolomic screening of the V1 tissue. **b**–**g** The effects of 30-min 40 Hz flickering on the intracellular metabolic changes, including major adenosine-generating, adenosine-degrading, and efflux pathways in V1 assessed 30 min after flickering cessation by the targeted UHPLC-MS/MS analysis. Adenosine (Ado, **b**), ATP (**c**), ADP (**d**), AMP (**e**), cAMP (**f**), and NAD^+^ (**g**). *P < 0.05 for the 40 Hz-treated vs normal light groups at 30 min; **P < 0.01 when comparing 40 Hz vs normal light groups at 240 min. **h** The effect of 40 Hz light flickering on the level of IMP, a main component in the salvage/de novo pathway, in V1. **P < 0.01 when comparing 40 Hz vs normal light groups at 240 min. **i**–**l** The effect of 40 Hz light flickering on metabolites involved in SAM-mediated transmethylation, including SAM (**i**), SAH (**j**), Hcy (**k**), and Met (**l**) in V1. *P < 0.05 when comparing 40 Hz with normal light groups at 240 min in (**l**), **P < 0.01 when comparing 40 Hz group at 30 min or 40 Hz group at 240 min with the groups exposed to normal light (**l**). **m**–**o** The effect of 40 Hz light flickering on the adenosine degradation product, inosine (by UHPLC-MS/MS analysis; **m**) and on mRNA expression of adenosine-degrading enzymes ADK (**n**) and ADA (**o**) in V1. *P < 0.05 when comparing the 40 Hz group with the normal light group at 240 min. **p**–**r** The effects of 40 Hz light flickering on the density and mRNA expression of *AMPK-α1* (**p**) and *AMPK-α2* (**q**) (by qPCR analysis) and on AMPK phosphorylation (by western blot analysis; **r**) in V1. *P < 0.05, Student’s *t*-test; comparing the 40 Hz group with the normal light group at 240 min after flickering cessation. **s** Schematic showing that 40 Hz flickering is mainly associated with the stepwise ATP dephosphorylation for energy metabolism, rather than SAM-mediated transmethylation or S-adenosylhomocysteine hydrolysis, or the salvage/de novo purine synthesis pathways.

We further investigated the specific pathways responsible for intracellular adenosine generation in response to 40 Hz flickering, including stepwise ATP dephosphorylation, SAM-mediated transmethylation, salvage/de novo purine synthesis, and impaired adenosine metabolism via adenosine deaminase (ADA). We performed targeted UHPLC-MS/MS and qPCR analysis to specifically assess purine metabolites and the expression of key enzymes involved in purine intracellular metabolism (Fig. 4a). (a) Consistent with the cascade amplification of adenosine generation from stepwise ATP dephosphorylation driven by increased energy consumption, targeted UHPLC-MS/MS analysis revealed a rise in the total concentrations of adenosine, ADP, and AMP but without changes in ATP levels in V1 assessed 240 min after 40 Hz flickering stimulation (Fig. 4b–e). There were also no changes in the levels of cAMP and nicotinamide adenine dinucleotide (NAD) (Fig. 4f, g), indicating that these adenosine-generating pathways were not likely to contribute. (b) The targeted UHPLC-MS/MS analysis also revealed a decrease in inosine monophosphate (IMP) levels after 40 Hz flickering stimulation (Fig. 4h), suggesting that the salvage/de novo purine synthesis pathway was an unlikely contributor to the increased adenosine generation. (c) Intracellular adenosine can also be generated by the hydrolysis of S-adenosylhomocysteine (SAH) into adenosine and homocysteine (Hcy) via SAH hydrolase (SAHH) within the S-adenosylmethionine (SAM)-dependent transmethylation pathway.^15–17^ The targeted UHPLC-MS/MS analysis revealed no changes in SAM, SAH, or methionine (Met) associated with increased Hcy levels after 40 Hz flickering (Fig. 4i–l). Thus, it is unlikely that the increased generation of intracellular adenosine is due to the involvement of SAM-dependent transmethylation. (d) The UHPLC-MS/MS analysis showed elevated levels of inosine (Fig. 4m) and qPCR analysis revealed an increase in *ADA* mRNA, but not astrocytic adenosine kinase (*ADK*) mRNA (Fig. 4n, o), suggesting a compensatory increase in adenosine metabolism through deamination of adenosine to inosine via ADA (Fig. 4s) and not through phosphorylation of adenosine to AMP by ADK (Fig. 4s). This indicates that reduced adenosine metabolism is not a contributing factor to intracellular adenosine accumulation after 40 Hz flickering. Taken together, the UHPLC-MS/MS and qPCR analyses suggest that the increased intracellular adenosine generation induced by 40 Hz flickering is mainly associated with the stepwise ATP dephosphorylation for energy metabolism, rather than SAM-mediated transmethylation or SAH hydrolysis, or the salvage/de novo purine synthesis pathways, or the impaired adenosine metabolism via ADA (Fig. 4s). Altered energy metabolism with increased production of endogenous adenosine is critically dependent on AMPK, a key metabolic controller.^38^

To further confirm the involvement of increased energy metabolism in extracellular adenosine generation, we investigated the time (30 and 240 min after exposure) dependence of light flickering on phosphorylated AMPK by western blot analysis and *AMPK* mRNA by qPCR analysis in V1. After 40 Hz light flickering, we observed an increase in *AMPK-α1* mRNA, but not *AMPK-α2*, in V1 at 240 min compared to control mice exposed to normal light (Fig. 4p, q). Similarly, 240 min after light flickering cessation, we observed a significant increase in phosphorylated AMPK in response to 40 Hz light flickering (Fig. 4r). Thus, the increased expression and phosphorylation of AMPK may contribute to the delayed and sustained increase in extracellular adenosine levels after light flickering.

### Brief 40 Hz light flickering promotes sleep onset and maintenance without affecting slow-wave activity power in mice

**Fig. 5:**
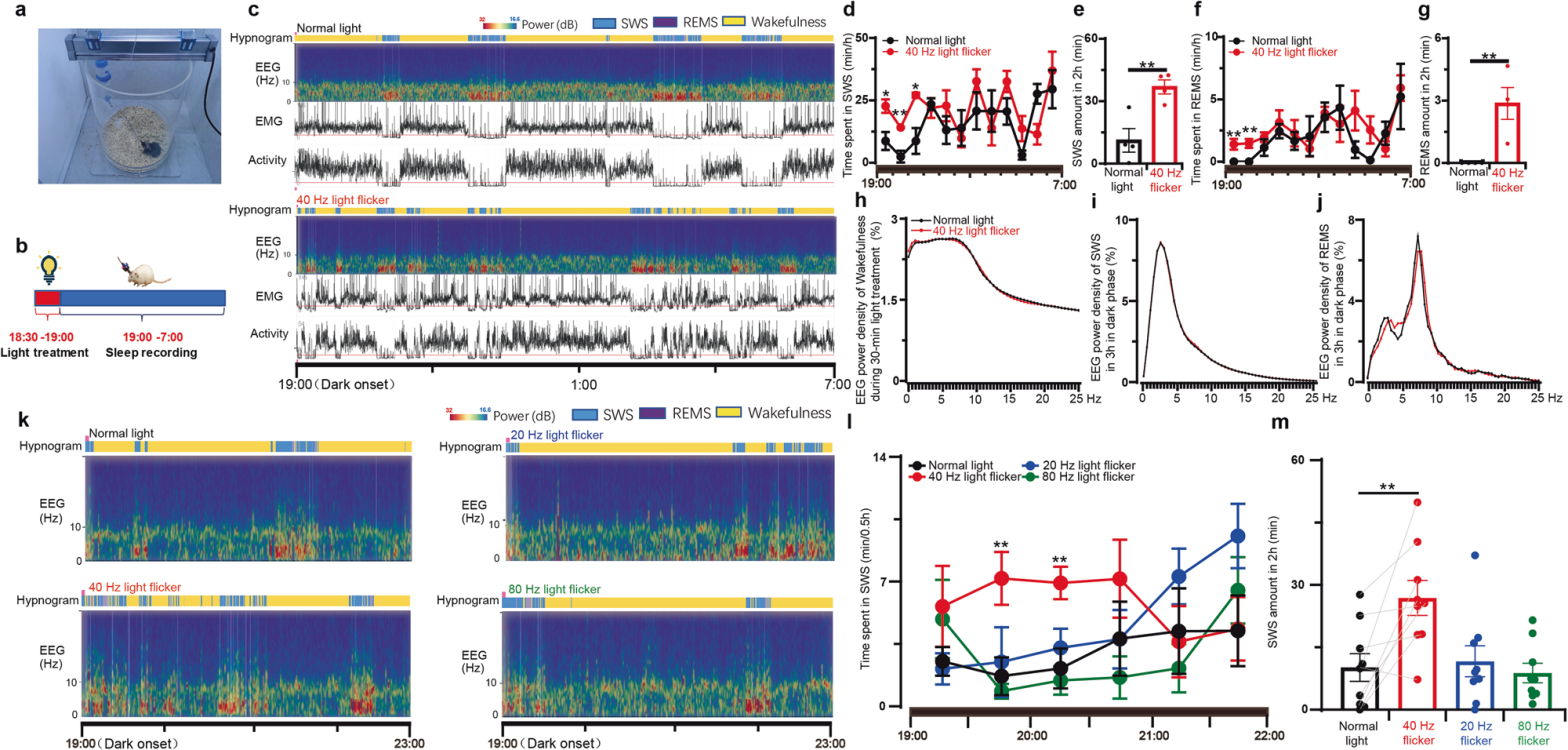
Light flickering at 40 Hz induces sleep in a frequency-dependent manner. **a** Paradigm of construction of light treatment and sleep recording system for mice. **b** Schedule for light treatments and sleep recordings. **c** The hypnogram, EEG spectrum, EMG, and locomotor activity in mice exposed to normal light and 40 Hz light flickering, respectively. **d**, **f** The time course of SWS and REMS in mice throughout the dark phase after exposure to normal light or 40 Hz light flickering treatment 30 min before the dark phase, respectively (*n* = 4/group); *P < 0.05; **P < 0.01, mean ± SEM, 40 Hz vs normal light, assessed by two-way ANOVA and Student’s *t*-test. **e**, **g** The amoun*t* of SWS and REMS during the initial 2 h after exposure of mice to normal light or 40 Hz light flickering 30 min before the dark phase (*n* = 4/group); *P < 0.05, 40 Hz vs normal light, assessed by Student’s *t*-test. **h** Superimposable power density of the wakefulness EEG spectra in mice during 30 min normal light or 40 Hz light flickering treatments (*n* = 4/group). **i** Superimposable power density of the SWS EEG spectra in mice after either normal light or 40 Hz light flickering treatments (*n* = 4/group). **j** Superimposable power density of the REMS EEG spectra in mice after either normal light or 40 Hz light flickering treatments (*n* = 4/group). **k** Representative hypnograms and EEG spectra of mice after treatments with light flickering at 20, 40, and 80 Hz, respectively. **l** The time course of SWS during 30 min blocks in the first 3 h after illumination at 20, 40, and 80 Hz frequencies. **P < 0.01 indicating significance at 20, 40, and 80 Hz compared to normal light using Student’s *t*-test (*n* = 9/group). **m** The amount of SWS during the following 2 h after exposure to normal light or 20, 40, and 80 Hz light flickering treatments 30 min before the dark phase (*n* = 9/group).

To consolidate the adenosine hypothesis as the neurochemical basis for the biological effects of 40 Hz light flickering, we evaluated the effect of 40 Hz visual pulses on sleep, since adenosine is a well-known physiological regulator of homeostatic sleep needs. We investigated the effects of 40 Hz light flickering on sleep induction by delivering light stimuli at 10:00 AM (early light cycle) or 6:30 PM (the end of the light cycle) for 30 min and recorded EEG/EMG/locomotion for the next 12 h. On day 1, mice were exposed to normal light, and we recorded EEG/EMG/locomotion. On day 2, we exposed mice to 40 Hz light flickering early at 10:00 AM or 6:30 PM for 30 min and then recorded EEG/EMG/locomotion for the next 12 h (Fig. 5a, b; Supplementary information, Fig. S4a). By comparing the sleep duration on day 2 to that on day 1, we observed a significant increase in the sleep amount when we delivered 40 Hz flickering at 6:30 PM (Fig. 5c–g). However, 40 Hz light flickering at 10:00 AM was not effective probably due to a relatively higher sleep baseline during the light phase (Supplementary information, Fig. S4b, c). During the first 3 h of the dark phase, the previous 40 Hz flickering significantly increased the total duration of sleep by enhancing both slow-wave sleep (SWS) (Fig. 5d, e, increased by 2.3-fold, mean ± SEM from 11.18 ± 5.66 to 36.87 ± 3.338 min) and rapid-eye-movement sleep (REMS) (Fig. 5f, g, mean ± SEM from 0 ± 0 to 2.87 ± 0.76 min), compared to mice exposed to normal light. Moreover, we confirmed the frequency specificity of the somnogenic effect of light flickering by demonstrating that exposure to visual flashing at 20 or 80 Hz did not alter the sleep pattern (Fig. 5k–m). The detailed analysis of sleep amount at 30-min intervals revealed that the effect of light flickering frequency on sleep was not immediate but became evident and sustained for 2 h after flickering cessation (Fig. 5l). These findings are consistent with the frequency-specific effect on extracellular adenosine generation (Fig. 1e, f). The correspondence between the frequency-specific effect on extracellular adenosine production and sleep amount suggests that the sleep-promoting effect of 40 Hz flickering is due to increased extracellular adenosine signaling in the brain. Notably, there was no significant difference in the delta power density of wakefulness during light treatment (Fig. 5h), SWS during the dark phase and theta power density of REMS during the dark phase between mice exposed to normal light and those exposed to 40 Hz flickering, respectively (Fig. 5i, j). This observed lack of an impact of 40 Hz light flickering on SWS delta power density suggests that it may be a potential treatment for insomnia, as it promotes sleep without side effects of daytime fatigue and drowsiness.

### The 40 Hz flickering-induced sleep-promoting effect requires ENT2 but not ENT1 activity

**Fig. 6:**
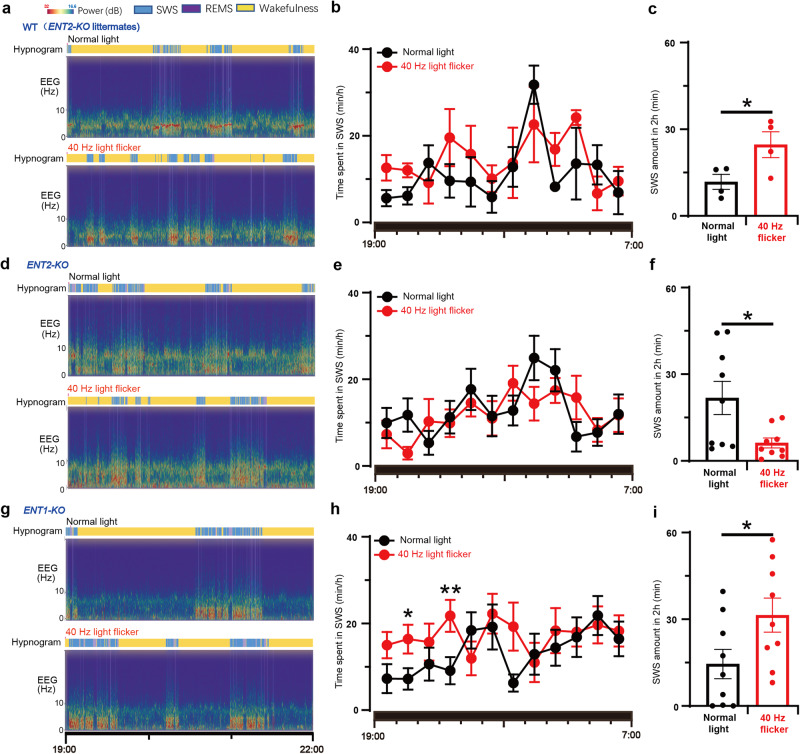
The sleep-promoting effect of 40 Hz light flickering is mediated by ENT2, but not ENT1, in mice. **a** Representative hypnograms and EEG spectra of WT littermates after treatments with normal light and 40 Hz light flickering, respectively. **b** The time course of SWS during the entire dark phase after light flickering was recorded and analyzed in WT littermates. **c** The amount of SWS in the first 2 h was compared between WT littermate mice subjected to 40 Hz flickering and normal light, and statistical significance (*P < 0.05) was evaluated using Student’s *t*-test (*n* = 4). **d** Representative hypnograms and EEG spectra of *ENT2*-KO mice after treatments with normal light and 40 Hz light flickering, respectively. **e** The time course of SWS during the entire dark phase after light flickering was recorded and analyzed in *ENT2*-KO mice. **f** The amount of SWS in the first 2 h was compared between *ENT2*-KO mice subjected to 40 Hz flickering and normal light, and statistical significance (*P < 0.05) was evaluated using Student’s *t*-test (*n* = 9). **g** Representative hypnograms and EEG spectra of *ENT1*-KO mice after treatments with normal light and 40 Hz light flickering, respectively. **h** The time course of SWS during the entire dark phase after light flickering was recorded and analyzed in *ENT1*-KO mice. **i** The amount of SWS in the first 2 h was compared between *ENT1*-KO mice subjected to 40 Hz flickering and normal light, and statistical significance (*P < 0.05; **P < 0.01) was evaluated using Student’s *t*-test (*n* = 9). The time course of SWS was analyzed using two-way ANOVA, while the difference in the total sleep amount each hour was analyzed with a paired Student’s *t*-test. *P < 0.05 was considered significant. Data are presented as mean ± SEM.

To further confirm the crucial role of extracellular adenosine signaling via ENT2 in mediating the sleep-promoting effect of 40 Hz flickering, we conducted sleep profile recordings (EEG and EMG) in mice deficient in either ENT1 or ENT2 after exposure to 40 Hz flickering for 30 min. In WT mice (*ENT2*-KO littermates), 40 Hz flickering stimulation increased SWS amount during the first 2 h by 1.1-fold (mean ± SEM from 11.77 ± 2.59 to 24.65 ± 4.49 min) (Fig. 6a–c). However, the sleep-inducing effect of 40 Hz light flashing was abolished in *ENT2*-KO mice and even lower than the normal light treatment, indicating that ENT2 is critical not only for mediating the sleep-inducing effect of 40 Hz light flickering but also for spontaneous sleep/wakefulness regulation (Fig. 6d–f). In contrast, 40 Hz flickering still significantly increased SWS in *ENT1*-KO mice, with the total amount of sleep increasing by 1.5-fold (mean ± SEM from 14.50 ± 5.08 to 31.43 ± 5.91 min) during the first 2 h (Fig. 6g–i), consistent with the lack of effect of *ENT1*-KO on extracellular adenosine levels. These findings support the conclusion that maintenance of extracellular adenosine activity via ENT2, but not ENT1, is required for the sleep-promoting effect of 40 Hz light flickering.

### 40 Hz light flickering promotes sleep via V1 adenosine signaling

**Fig. 7:**
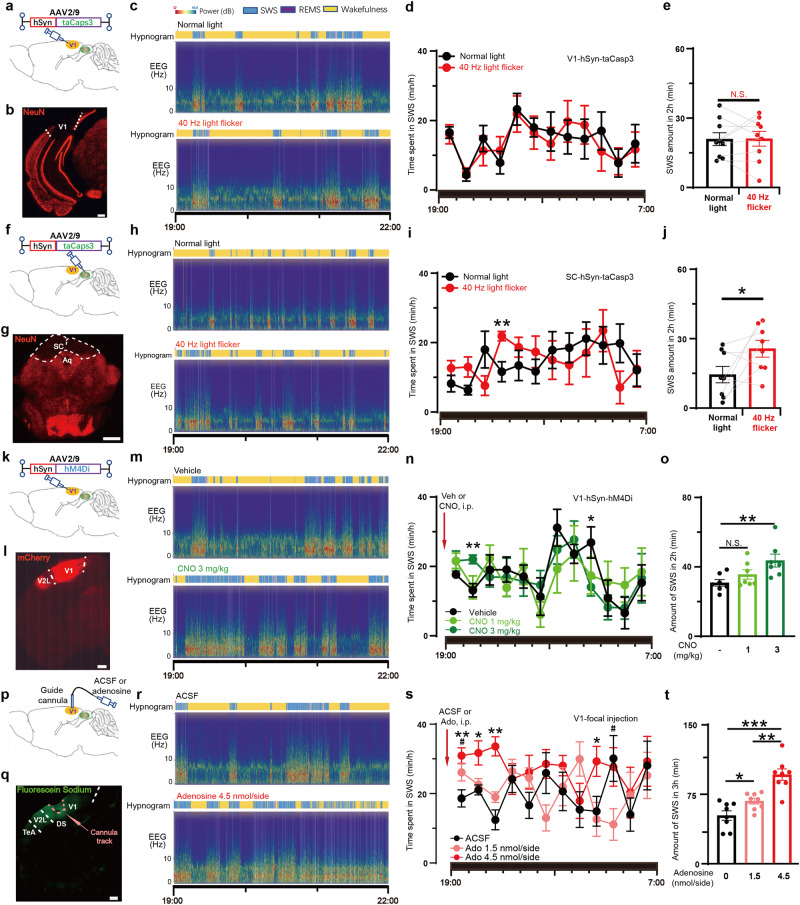
40 Hz light flickering promotes sleep via V1 adenosinergic signaling. **a**, **f** Schematic representation of AAV2/9-hSyn-taCaspase3 injection into V1 and SC, respectively. **b**, **g** Neural ablations of V1 and SC were visualized by fluorescence staining of NeuN, respectively. Scale bar: 500 μm (**b**); 1 mm (**g**). **c**, **h** Representative hypnograms and EEG spectra of mice with V1 or SC neural ablation after treatments with normal light and 40 Hz light flickering, respectively. **d**, **i** The time course of SWS during the entire dark phase after light treatments was recorded and analyzed in V1- and SC-neuron ablated mice, respectively (**P < 0.01). **e**, **j** The amount of SWS in the first 2 h was compared in V1- and SC-neuron ablated mice subjected to 40 Hz flickering and normal light, respectively, and statistical significance (*P < 0.05; **P < 0.01) was evaluated using Student’s *t*-test (V1 ablated, *n* = 9; SC ablated, *n* = 8). **k** Schematic representation of AAV2/9-hSyn-hM4Di-P2A-mCherry injection into V1. **l** Spontaneous fluorescence of mCherry indicates AAV expression in V1. Scale bar: 500 μm. **m** Representative hypnograms and EEG spectra of mice after intraperitoneal injections with the vehicle and CNO 3 mg/kg, respectively. **n** The time course of SWS during the entire dark phase after vehicle and CNO injections was recorded and analyzed (*P < 0.05; **P < 0.01). **o** The amount of SWS in the first 2 h was compared in mice injected with vehicle, CNO 1 mg/kg and 3 mg/kg, respectively, and statistical significance (**P < 0.01) was evaluated using one-way ANOVA (*n* = 7). **p** Schematic representation of focal injection of adenosine into V1. **q** Spontaneous fluorescence of fluorescein sodium at 1 h after focal injection into V1 indicates diffusion area of water-soluble substances. Scale bar: 500 μm. **r** Representative hypnograms and EEG spectra of mice after bilateral focal injections of ACSF and adenosine at 4.5 nmol/side, respectively. **s** The time course of SWS during the entire dark phase after bilateral focal injections of ACSF and adenosine was recorded and analyzed. **t** The amount of SWS in the first 3 h was compared mice with bilateral focal injections of ACSF, adenosine at 1.5 nmol/side and 4.5 nmol/side into V1, respectively, and statistical significance (*P < 0.05; **P < 0.01) was evaluated using one-way ANOVA (*n* = 8).

To investigate the role of V1 in 40 Hz light flickering-induced sleep, we measured sleep after ablation of neurons in V1, and SC (which had a similar increase in adenosine after 40 Hz flicker light stimulus, Fig. 1b, c), by using AAV2/9-hSyn-taCaspase3 virus (Fig. 7a, b, f, g; Supplementary information, Fig. S7a–f). Pretreatment of 40 Hz light flickering in the last 30 min of light period failed to induce sleep in V1-neuron-ablated mice (Fig. 7d, e, normal light: 20.92 ± 2.78 min; 40 Hz flickering: 21.08 ± 3.20 min). However, in the SC-neuron-ablated mice 40 Hz light flickering only resulted in a non-significant increase of SWS (Fig. 7i, j, normal light:14.48 ± 3.53 min; 40 Hz flickering: 25.65 ± 3.60 min) in the first 2 h after dark onset. These data suggest that V1 is a key nucleus in mediating 40 Hz light flickering-induced sleep while SC’s involvement is relatively insignificant.

We hypothesize that the increased adenosine acts on A_1_ receptors (A_1_R, a Gi coupled GPCR), which is the predominant adenosine receptor subtype in V1 neurons to inhibit neural activities. We silenced V1 neurons using DREADD (designer receptors exclusively activated by designer drugs). Two weeks after AAV2/9-hSyn-hM4Di-mCherry injections into V1 to selectively express a modified human muscarinic receptor in neurons, vehicle or CNO (Clozapine N-oxide) at 1 mg/kg and 3 mg/kg were administered to mice by intraperitoneal injections (Fig. 7k, l; Supplementary information, Fig. S7 g, h). The time course and 2 h-amount of SWS of CNO injection at 1 mg/kg (35.58 ± 2.71 min) was similar to vehicle injection (30.79 ± 1.90 min) (Fig. 7m–o). The CNO at 3 mg/kg (43.62 ± 3.72 min) significantly increased SWS and REMS for 2 h after injections, indicating that reducing activity of V1 neurons has sleep-promoting effects (Fig. 7m–o; Supplementary information, Fig. S6a, b).

To confirm the sleep-promoting effects of adenosine on V1 neurons, we directly applied adenosine to V1. Injection cannulas were implanted for ten days (Fig. 7p, q), and adenosine (1.5 or 4.5 nmol/side) or artificial cerebrospinal fluid (ACSF) was microinjected into V1 on days 11, 14, and 17 at 6:30 PM. We monitored the sleep patterns of the mice for the next 12 h, from 7:00 PM to 7:00 AM. The injection of adenosine at both doses resulted in a significant increase in the amount of SWS (ACSF group: 52.11 ± 5.11 min; adenosine 4.5 nmol/side: 96.24 ± 6.14 min; adenosine 1.5 nmol/side: 67.64 ± 3.18 min; mean ± SEM) during the first 3 h (Fig. 7r–t) with the higher adenosine dose producing more SWS (Fig. 7t). These data provide direct evidence that the local elevation of extracellular adenosine levels in V1 is sufficient to promote SWS and that the visual cortex plays a crucial role in mediating 40 Hz flickering-induced sleep via adenosine signaling.

### 40 Hz light flickering reduces sleep latency and wake time after sleep onset while increasing total sleep time (TST) and efficiency in children with insomnia

**Fig. 8:**
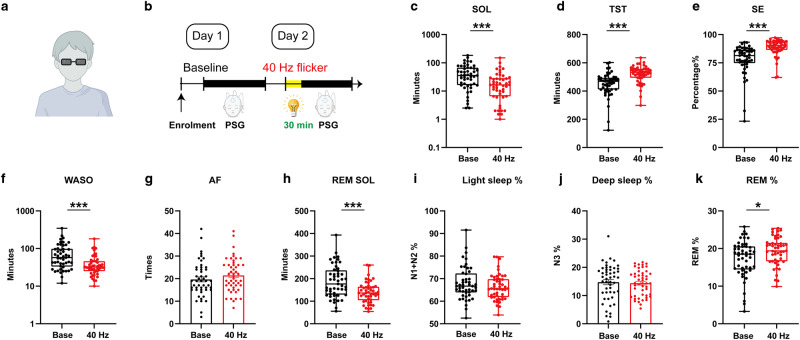
40 Hz light flickering improves sleep onset and maintenance among children with insomnia. **a** Schematic figure for 40 Hz flickering stimulation. **b** Illustrations of the study design, in which patients were enrolled for two consecutive days: day 1 served as a baseline; day 2 involved 30 min of 40 Hz flickering stimulation before sleep. **c** 40 Hz flickering significantly reduced SOL (*W* = –767, *P* < 0.001). **d** 40 Hz flickering significantly increased TST (*W* = 905, *P* < 0.001). **e** SE was also improved by 40 Hz flickering (*W* = 1055, *P* < 0.001). **f** WASO was remarkably reduced by 40 Hz flickering stimulation (*W* = –924, *P* < 0.001). **g** AF remained unchanged (*t*_(48)_ = 1.649, *P* = 0.106) after 40 Hz flickering stimulation. **h** REM SOL was also reduced by 40 Hz flickering (*t*_(48)_ = 4.521, *P* < 0.001). **i**, **j** The percentage of light (**i**) and deep sleep (**j**) remained unchanged (*W* = –322, *P* = 0.100; *t*_(48)_ = 0.510, *P* = 0.612). **k** An increase in REMS percentage was detected (*W* = 447, *P* = 0.025) after 40 Hz flickering stimulation. A paired *t*-test or Wilcoxon matched-pairs signed rank test was conducted based on the distribution types of the paired difference. N1 + N2, light sleep; N3, deep sleep. *P < 0.05, **P < 0.01, ***P < 0.001; Data are presented as mean ± SEM or median (lower quartiles–upper quartiles).

Finally, we conducted a self-controlled clinical study to assess the effectiveness of 40 Hz light flickering in promoting sleep onset and maintenance in 49 children with insomnia symptoms. The children admitted in this study had an average age of 9.63 ± 3.28 years and body-mass index (BMI) of 15.60 (14.70–18.00 kg/m^2^), both sexes balanced (44.90% female) (also see Table 1). This specific patient population was selected for its relatively milder insomnia and thus presumably sensitive to 40 Hz light flickering treatment. Because human sleep polysomnography (PSG) was generally monitored at night, we conducted this study by delivering 40 Hz light flickering at 8:30 PM for 30 min. We delivered light flickering stimulation by wearing the glasses with 40 Hz light flickering at 2000 lux for tolerability in children but still producing comparable light intensity (0.58 mW/cm^2^ at 2 cm distance). Indeed, this protocol did not affect IOP (Supplementary information, Fig. S1a), the choroidal vascular index, total choroidal area, luminal area, and retinal thickness of the adult volunteer eye (Supplementary information, Fig. S1b–f). No complaints of discomfort were recorded among participants (Supplementary information, Tables S1 and S2). The patients underwent sleep adjustment at the sleep center and their sleep parameters (including EEG and EMG) were monitored over two days. On the first day, sleep parameters were assessed for the patients’ baseline activities. On the second day of the study, the patients were exposed to 40 Hz light flickering for 30 min (8:30–9:00 PM) and their sleep parameters were recorded during the entire night (Fig. 8a, b). Analysis of the primary endpoint by comparing day 2 (40 Hz light flickering) with day 1 (baseline with normal light exposure) using PSG analysis revealed that 40 Hz light flickering significantly reduced sleep onset latency (SOL), which is the duration from light off to sleep onset (*W* = –767, *P* < 0.001), as depicted in Fig. 8c and Table 2. Furthermore, analysis of the secondary endpoints using PSG also showed that 40 Hz light flickering had a positive effect on TST (*W* = 905, *P* < 0.001, Fig. 8d and Table 2). Sleep efficiency (SE, total sleep time/total time in bed) was similarly enhanced by 40 Hz light flickering (*W* = 1055, *P* < 0.001, Fig. 8e and Table 2). Light flickering at 40 Hz reduced wake time after sleep onset (WASO, minutes of wake after sleep onset) (*W* = –924, *P* < 0.001, Fig. 8f and Table 2). We noted that 40 Hz light flickering had no significant effect on arousal frequency (AF, defined as the number of awakenings during the night; *t*_(48)_ = 1.649, *P* = 0.106; Fig. 8g and Table 2). In addition, 40 Hz light flickering also shortened REM sleep onset latency (REM SOL, measured in minutes between sleep onset and the first epoch of REM sleep; *t*_(48)_ = 4.521, *P* < 0.001, Fig. 8h and Table 2). Notably, the percentage of light sleep (Fig. 8i and Table 2) or deep sleep (Fig. 8j and Table 2) remained unchanged after 40 Hz light flickering (*W* = –322, *P* = 0.100; *t*_(48)_ = 0.510, *P* = 0.612). We found that 40 Hz flickering increased REM sleep percentage (*W* = 447, *P* = 0.025, Fig. 8k). Taken together, 40 Hz light flickering for 30 min (between 8:30 PM and 9:00 PM) was found to enhance sleep onset and maintenance in children with insomnia.

**Table 1:** The characteristics of children with insomnia symptoms

Characteristics	
Age	9.63 ± 3.28 years
Female number	22 (44.9% of all participants)
BMI (kg/m^2^)	15.60 (interquartile range is 3.30)

**Table 2:** Effects of 40 Hz flicker on sleep parameters in children with insomnia symptoms

	Baseline (Day 1)	40 Hz (Day 2)	*P*-value
SOL (min)	35.5 (16.3–66.8)	16.5 (6.5–28.5)	<0.001***
TST (min)	468.5 (411.0–494.0)	532 (494.3–556.3)	<0.001***
SE (%)	81.40 (74.7–86.5)	90.4 (86.4–93.9)	<0.001***
WASO (min)	57.50 (33.3–98.5)	32(25.0–46.0)	<0.001***
AF (*n*)	19.7 ± 1.1	21.5 ± 1.1	0.106
REM SOL (min)	187.6 ± 10.21	138.7 ± 6.6	<0.001***
Light sleep, N1 + N2 (%)	66.7 (63.9–72.3)	65.6 (61.9–69.8)	0.100
Deep sleep, N3 (%)	14.8 ± 0.9	14.4 ± 0.7	0.612
REM (%)	18.5 (14.5–20.6)	19.4 (16.6–21.5)	0.025*

*n* = 49. Data are presented as mean ± SEM or median (lower quartiles–upper quartiles).

*N1* *+* *N2* light sleep, *N3* deep sleep.

*P < 0.05, **P < 0.01, ***P < 0.001.

## Discussion

Our findings established cortical adenosine signaling as the neurochemical basis responsible for the sleep-inducing effects of 40 Hz light flickering. Our data describe a phenomenon of photo-electro-chemical coupling where 40 Hz light flickering triggered neural rhythmic fluctuations in LFPs, specifically in the gamma range. This energy-demanding process leads to heightened energy metabolism resulting in increased adenosine generation.^15^ The relationship between specific visual pattern stimulus selectively enhancing LFP oscillations in the gamma range is tightly linked to mitochondrial activity in the brain,^12–14^ accompanied by increased regional cerebral blood flow in healthy individuals.^6,39,40^ The observed changes in gamma oscillations and increase in extracellular adenosine levels occurred predominantly in the primary visual cortex. The increase in extracellular adenosine levels was induced by an increase in AMPK phosphorylation. AMPK is a key sensor and controller of cellular energy metabolism and a regulator of sleep quality.^41,42^ The intrinsic link between the high cellular metabolism imposed by gamma oscillation and adenosine generation, together with the multiple physiological effects of adenosine as a dual neuromodulator, and homeostasis regulator, uniquely position the adenosine signaling system to serve as the unique and pivotal neurochemical basis for the biological effects of 40 Hz light flickering. The present study also identified ENT2 as a key molecular regulator of the therapeutic effects of 40 Hz light flickering effect on sleep control since the genetic deletion of ENT2, but not ENT1, abolished the frequency-specific effects of light flickering on both extracellular adenosine generation and the induction and maintenance of sleep. The critical role of ENT2 in the generation of extracellular adenosine matches recent findings that neuronal activity-induced outflow of adenosine from neurons after high-frequency stimulation in the HPC is ENT-dependent.^33,36,43^ The greater importance of ENT2 compared to ENT1 may be attributed to its larger capacity to transport adenosine, with four times higher expression in neurons and astrocytes, allowing larger increases of extracellular adenosine levels in conditions such as light flickering.^37,44^

The present discovery that 40 Hz light flickering can specifically evoke an endogenous homeostatic regulatory mechanism operated by increased adenosine signaling, provides a novel, promising and non-invasive approach to insomnia treatment. Our experimental protocol delivered the 40 Hz light flickering during the end of the light cycle and recorded sleep 3 h during the dark cycle. Mice are nocturnal animals and hence, are more active during the dark cycle. We were able to measure an increase in time spent in SWS and REMS, demonstrating the effect of 40 Hz light flickering on sleep induction. Importantly, 40 Hz light flickering stimulation did not affect the ratio of deep to light sleep in children with insomnia, suggesting an improved SE without significant sleep rebound or side effects.^20,45^ This is further supported by the lack of effect on delta power density in mice. Our study found that the luminance intensity (4000 lux, 1.22 mW/cm^2^ at 20 cm distance in mice and 2000 lux, 0.58 mW/cm^2^ at 2 cm distance in humans), which is higher than that used in other 40 Hz studies (usually from 450–700 lux),^4,46^ is a critical factor to produce robust and sustained increase of adenosine level in the cortex. Our protocol of brief (30 min) light stimulus is well within the safety limit of 5000 lux light within 10 h^3,31,32^ and did not produce any alteration of retina structures and functions by optical coherence tomography (OCT) and blood assays in mice and humans. Our novel and non-invasive approach overcomes the bottleneck of adenosine drug-based therapies for insomnia with poor permeability of the central nervous system and adverse cardiovascular and liver effects.^16,19^

Traditionally, sleep-wake regulation is achieved by the subcortical (particularly, the brain stem and hypothalamus) network while the cortex is thought to be responsible for generating homeostatic sleep pressure and exerting top-down feedback connections to the subcortical region. However, this view has been challenged recently^47–50^ with the demonstration of the shared and structured global patterns of cortical activity^50,51^ and modulation of sleep state switch by optogenetic inhibition of the REM-like pattern in the mouse occipital cortex.^50,51^ Our analysis established the adenosine signaling in V1 neurons as the critical loci whereby 40 Hz light flickering promotes sleep. Our data showed that (i) specific lesioning of V1 (but not SC) neurons abolished the somnogenic effect of 40 Hz light flickering; (ii) chemogenetic inhibition (hM4Di-DREADD) of V1 neurons promotes sleep; (iii) the focal infusion of adenosine into V1 reproduces the somnogenic effects of 40 Hz light flickering. Notably, while adenosine control of spontaneous/physiological sleep is achieved by adenosine signal in the basal forebrain^20^ and involves relatively milder change of adenosine level (∆*F*/*F* = 2%–4%),^21^ pharmacological control of sleep-wake cycle by adenosine involves strong inhibition of V1 neurons by 40 Hz light flickering (with robust adenosine increase (∆*F*/*F* > 20%) or chemogenetic inhibition). Thus, while the visual cortex may be not involved significantly in spontaneous sleep/wake cycles,^52,53^ strong inhibition of V1 (via adenosine) is sufficient to break spontaneous routines and to trigger a switch between sleep/wakefulness vigilance stages. Whether V1 adenosine acts at adenosine A_1_ receptor to mediate the sleep-promoting effects of 40 Hz light flickering awaits for further investigation in future studies. Thus, adenosine signaling serves as a gateway for both physiological and pharmacological transitions from wakefulness to sleep by directly modulating activity in the BF and visual cortex respectively.

This identification of extracellular adenosine as the molecular and neurochemical underpinning of 40 Hz flickering fills the critical gap between bolstered neuronal activity (specifically gamma oscillation) and therapeutic effects in AD and ischemic pathologies such as beta-amyloid reduction and cognitive improvements that are controlled by the adenosine modulation system.^18^ For example, adenosine signaling can activate microglial cells^4,5^ and enhance vascularization in various tissues,^6^ including our recent demonstration that the A_2A_ receptor controls microglial activation^18,54^ and retinal vascularization.^55,56^ The induction of adenosine signaling through 40 Hz flickering is expected to boost microglial activation and vascularization in the cortex, increasing the clearance of β-amyloid species and improving cognition in AD mice. Furthermore, the protective effect of light flickering at 30–50 Hz against ischemia-induced hippocampal neuronal death and disruption of hippocampal CA1 low gamma oscillations^7^ can be partially attributed to increased extracellular adenosine levels, which suppress excessive excitatory transmission by acting as a gatekeeper mechanism via the adenosine A_1_ receptor.^18^ Thus, this study reveals a novel and non-invasive treatment for insomnia with potentially wider therapeutic implications for other neuropsychiatric disorders, attributed to the dual roles of adenosine as both a neuromodulator and homeostatic regulator. Our current data show specific sleep-inducing and sleep maintenance effects of 40 Hz flickering light. Additional clinical studies with more insomnia patients including adult insomnia will be required to understand the effectiveness of 40 Hz flickering light in the treatment of insomnia. 40 Hz flickering light therapy has good potential for the treatment of jetlag where travelers across time zones will need to sleep at a time that is against their normal circadian rhythm.

## Materials and methods

### Animals

All experimental protocols were approved by the Institutional Ethics Committee and followed the guidelines for Animal Use in Research and Education at Wenzhou Medical University, China (NO. wydw2021-0563). C57BL/6 WT mice were purchased from institute-approved vendors (Beijing Vital River Laboratory Animal Technology Co., Ltd.).

vGluT2-IRES-CRE mice JAX Strain (016963)^57^ and *Nt5e* (*CD73*) KO (*CD73*-KO) mice (JAX Strain 018986)^58^ were obtained from Jackson Laboratory. *ENT1*-KO mice, *ENT2*-KO mice,^36^ and GRAB_ATP1.0_ knock-in (GRAB_ATP1.0_-KI) mice were generated using CRISPR/Cas9 technology by Beijing Biocytogen and kindly provided by Dr. Yulong Li’s laboratory.^36^ The mice were housed in an enriched environment with ad libitum access to food and water under a 12-h light/12-h dark cycle. Mice with implants for EEG/EMG recording, optogenetic manipulation, and fiber photometry recording were housed individually.

### Virus injection and cannula implantation

The mice were anesthetized with isoflurane and head-fixed in a stereotaxic apparatus (Kopf, USA) to expose the skull and drill a hole for injection and implantation. (i) A craniotomy was performed on top of V1 and a 0.5–1 mm-thick glass pipette was used to inject (20 nL/s) virus (0.2–0.4 µL) at the following coordinates (V1:AP: –3.6 mm, ML: ±2.4 mm, DV: –0.6 mm; SC:AP: –3.8 mm, ML: ±0.7 mm, DV: –1.2 mm; mPFC:AP: +1.96 mm, ML: ±0.36 mm, DV: –1.65 mm; HPC:AP: –2.4 mm, ML: ±1.70 mm, DV: –1.30 mm; DMS:AP: +1.2 mm, ML: ±1.25 mm, DV: –2.6 mm; BF:AP: +0.3 mm, ML: ±1.05 mm, DV: –4.75 mm; NAc:AP: +1.20 mm, ML: ±1.25 mm, DV: –3.9 mm; VTA:AP: −3.40 mm, ML: ±0.4 mm, DV: –4.0 mm) from the cortical surface using KDS LEGATO 130 (KD Scientific, China). The following AAV viruses were used in the current study: AAV2/9-hSyn-GRAB_Ado1.0_ (titer ≥ 1 × 10^13^ vg/mL) and AAV2/9-GfaABC1D-GRAB_Ado1.0_, which was a gift from Dr. Yulong Li’s laboratory.^59^ AAV2/9-hSyn-GRAB_Ado-mut_ (titer ≥ 1 × 10^13^ vg/mL), AAV2/9-EF1a-flex-taCaspase3-TEVp (titer ≥ 1 × 10^13^ vg/mL), AAV2/9-hSyn-taCaspase3-T2A-TEVp (titer ≥ 5 × 10^12^ vg/mL), AAV2/9-hSyn-CRE(titer ≥ 1 × 10^13^ vg/mL), AAV2/9-mDlX-CRE (titer ≥ 5 × 10^12^ vg/mL), AAV2/9-hSyn-tdTomato (titer ≥ 5 × 10^12^ vg/mL), AAV2/9-hSyn-DIO-mCherry (titer ≥ 5 × 10^12^ vg/mL), AAV2/9-hSyn-hM4Di-P2A-mCherry (titer ≥ 5.26 × 10^12^ vg/mL) and AAV2/9-GfaABC1D-hPMCA2w/b-mCherry (titer ≥ 5 × 10^12^ vg/mL) were produced by the viral production facility of the BrainVTA company (China). Viruses were stored at −80 °C freezer until the day of infusion. (ii) After viral injection, a 0.5–1 mm diameter craniotomy was performed to implant one or two optical fibers with FC ferrules (200 μm in diameter, NA 0.37) (Inper, China) into the above injection site (for optogenetic manipulation) or in (for fiber photometry recordings) the injection site, and the implants were fixed to the skull with dental cement. Mice with virus injections and implants were allowed to recover for at least 3 weeks before fiber photometry recordings or optogenetic manipulation experiments. Viral expression and the position of fiber implants in each mouse were confirmed histologically after the termination of the experiments. (iii) For EEG recording, four stainless steel screws were implanted, with two located over the primary motor cortex (∼2 mm anterior to the bregma and 1 mm lateral to the midline) and the other two over the parietal cortex (approximately 2 mm posterior to the bregma and 1 mm lateral to the midline). (iv) For EMG recording, two stainless steel wires were implanted in the neck muscle. After attaching both EEG and EMG electrodes to a head-mounted device (Cat# 2631, Bio-Signal Technologies, China), the assembly was secured with dental cement. (v) To inject adenosine through the implanted cannula, two 26-gauge stainless steel guide cannulas (Cat# 62003, RWD Life Science, China) were stereotaxically implanted into V1 at AP –3.6 mm, left-right ±2.4 mm from the bregma, and DV –0.5 mm from the skull top. Matched dummy cannulas (Cat# 62003, RWD Life Science, China) were inserted into the guide cannulas after surgery and removed only during microinjections. Microinjections of ACSF or adenosine solutions were performed using a tubing-nested 10-µL Hamilton syringe (Cat# 80365, Hamilton, USA). After all surgical procedures, mice were housed individually in clear acrylic cages under controlled temperature, humidity, and lighting conditions (12-h light/12-h dark schedule), with ad libitum access to food and water.

### In vivo electrophysiology

#### Surgery

C57/BL6 mice aged 8–10 weeks were implanted with electrodes to record LFPs in V1. The surgical procedure, as previously described,^60^ involved anesthesia with isoflurane, head fixation in a stereotaxic apparatus (Kopf, USA), and exposing the skull. A cranial window (V1:AP: –3.6 mm, ML: ±2.4 mm, DV: –0.6 mm) was then prepared using a dental drill (RWD Life Science, China) on the left side for implantation. A 16-channel microwire array electrode (Cat# KD-MWA-16, Kedou, China) was inserted into V1 and grounded above the cerebellar hemispheres using two bone screws (Cat# 700-00122-00, RWD Life Science, China). The electrode was then secured in place with tissue glue (Cat#454, LOCTITE, Ireland) and dental cement (Cat# type II 2#, New Century Dental, China).

#### Recording

After a recovery period of 14 days following surgery, the mice were transferred to an electrophysiological recording chamber and allowed to acclimate for 20 min before recording began. When the experiment started, the mice were placed in a transparent and electromagnetically shielded box. Each mouse was connected to the recording system via Cereplex M (Cerebus, Blackrock Microsystems, USA), a 16-channel digitally programmable amplifier filtered (0.3 Hz–7.5 kHz), close to the animal’s head. The signals were sampled at 1000 Hz and refined with a low-pass filter at 250 Hz. The lamps generating different visual stimulation (DC, 10, 20, 40, and 80 Hz) were positioned face to face in front of the box. We isolated the stimulation and recording circuits to avoid any electromagnetic interference. We confirmed that there were no changes in the power of LFPs during visual stimulation in recordings from saline, dead mice (electrode implanted in V1), and negative contrast (electrode implanted in the primary motor cortex (M1)), which helped us exclude any potential photoelectric effect or other interference (data not shown). LFPs were initially recorded in V1 for 5 min under quiet and dark conditions, followed by 30 min of recording during visual stimulation with different frequencies (presented in random order for each mouse), and finally stopped after a 5-min post-stimulation period.

#### Analysis

The LFP data underwent a short-time Fourier transform with MATLAB R2020a and power within flickering frequency (specific frequency ±0.5 Hz) was calculated by integrating the power spectral density estimate using the rectangle method. We also normalized the LFP power within the flickering frequency to the max LFP power and normalized the adenosine level with a 40 Hz stimulus.

### Light flickering stimulation

Light flickering stimulation was performed using a previously described method.^4–6^ Before the experiment began, the mice were placed in a PVC case, similar to their home cage but without bedding, and kept under dim lighting for 1 h. Two LED bulbs were positioned on the long side of the cage opposite each other and controlled by a single circuit-control relay in a dark room. White LEDs, which emit a combination of visible wavelengths ranging from 390 to 700 nm, were used to provide five types of stimulation (dark, light, 20, 40, and 80 Hz) for 30 min, with stimulation consisting of 12.5 ms light on and 12.5 ms light off and using 60 W. The LEDs used in the experiment had a correlated color temperature (CCT) of 4000 K and were operated at various intensities, ranging from 50 to 6000 lux, including 50, 500, 1000, 2000, 3000, 4000, and 6000 lux. Before the onset of light, the irradiance was monitored by a dosimeter and the irradiance did not exceed ~1.5 mW/cm^2^ at the mouse position. Meanwhile, safety and tolerability assessments were performed continuously and illumination lasted a total of 30 min per day, applied for 14 contiguous days with 4000 lux (irradiance: 1.22 mW/cm^2^).

### Preparation and administration of drugs

Dipyridamole was dissolved in a 0.5% methylcellulose solution at 1.5 mg/mL. CNO was dissolved in saline into the required concentrations. Felodipine was dissolved in 2% DMSO with normal saline and covered to avoid light exposure. Felodipine and the vehicle (2% DMSO in saline) were intraperitoneally injected at a dose of 10 mg/kg body weight before 40 Hz flickering exposure. To prepare the cannula microinjection solution, adenosine was dissolved in ACSF (126 mM NaCl, 26 mM NaHCO_3_, 10 mM glucose, 2.5 mM KCl, 1.25 mM NaH_2_PO_4_, 2 mM MgCl_2_, and 2 mM CaCl_2_, pH 7.4) at the required doses (1.5 and 4.5 nmol/side) with an injection volume of 2 µL per side and injected slowly (~2 min) into the brain. All drug solutions were prepared approximately 30 min before injection. The injections were performed using a crossover design.

### Fiber photometry recording and analysis

To record fluorescence from the calcium, adenosine, or ATP sensor, we attached an optical fiber (core diameter 220 μm, numerical aperture 0.37, Inper, China) to the implanted ferrule through a ceramic sleeve and recorded the emission fluorescence using a commercial fiber photometry system (Thinker Tech Nanjing Biotech Co., Ltd., China). The excitation light reached the V1 after passing through the fiber jumper (Thinker Tech Nanjing Biotech Co., Ltd., China) and ceramic insert core of the mouse head, thereby exciting the green fluorescence signal. To record fluorescence signals, the laser beam from a 488-nm laser (OBIS 488LS; Coherent) was reflected by a dichroic mirror (MD498; Thorlabs), focused by a 10× objective lens (NA = 0.3; Olympus) and then coupled to an optical commutator (Doric Lenses). Fiber recordings were performed on freely moving mice during flicker visual stimulation, and no data were excluded. The mice were connected to the fiber photometry recording system after being habituated in a transparent test cage and the signals were recorded for 900 s as a baseline before flicker visual stimulation. To determine the effect of V1 neurons during flicker visual stimulation, a 10-s on, 10-s off pattern was adopted following injection of gCAMP6s virus sensors.

The signal from each continuous experimental trial was normalized to the average fluorescence using a MATLAB program developed by Thinkertech. Briefly, the raw signals were first adjusted to account for photo-bleaching by considering the overall trend before further analysis. To examine the response intensity of adenosine or ATP to light flickering stimulation, we obtained fluorescence change values (Δ*F*/*F*) that were calculated as (*F*–*F0*)/(*F0*–Voffset), where the baseline fluorescence signal (*F0*) was the average signal over a 300-s control time window. Peri-event plots were generated to display Δ*F*/*F* values, using polynomials to correct the baseline. In addition, the multi-color single-channel recording system is used to verify the experimental results of the multi-channel fiber photometry system. We then measured the area under the curve of the Δ*F*/*F* plot to quantify the response of light flickering stimulation. Mean values are presented as a peri-event plot, and shaded areas represent standard errors of the mean SEM.

### Polysomnographic recording and analysis

After at least one week of EEG/EMG implantation, the mice were housed individually in transparent barrels in a sound-proofed recording chamber with insulation and maintained on a 12-h light/12-h dark cycle with lights on at 7:00 AM. They were provided with ad libitum access to food and water. The mice were acclimated to the recording cable for at least 3 days before starting the recording process. The cortical EEG and EMG signals were amplified, filtered with a high-pass filter above 0.5 Hz and band-pass filtered between 5–45 Hz, digitalized at a 1000 Hz resolution using a tethered data acquisition system (Medusa, Bio-Signal Technologies, China), and synchronized with the infrared video. The power of the *δ* (0.5–4 Hz), *θ* (5–8 Hz), and *α* (9–14 Hz) bands, as well as *δ* band ratio and EMG/activity signal, were calculated and scored.^50,61^ For sleep behavior tests requiring light flickering treatment, light flickers of 20, 40, or 80 Hz at 4000 lux (illuminance, or irradiance was 1.22 mW/cm^2^, 50% duty cycle) were used. For sleep recording in the dark period, all light treatments were applied for 30 min just before the dark phase onset (the last 30 min in the light period), and animals were kept awake by introducing new nesting/food and hitting cage walls to ensure light perception. For sleep recording in the light period, all light treatments were applied for 30 min from the 3rd h after the light was on, and all animals were kept awake by introducing new nesting/food and hitting cage walls to ensure light perception.

The sleep stages in the recordings were scored by AI-driven software, Lunion Stage, developed by LunionData^62^ (https://www.luniondata.com/en/lunion_stage). The AI engine of Lunion Stage employs a convolutional neural network, which is a type of deep learning model. This model extracts spectrogram features from the EEG channel and movement features from the EMG channel. After being trained on manually labeled recordings by domain experts, the model’s accuracy is assessed on recordings from diverse sources, including different devices and subjects, and achieves an average accuracy exceeding 98%. The EEG/EMG data collected were analyzed in 4-s epochs, and three stages, namely slow-wave sleep, REMS, and wakefulness, were automatically recognized based on their spectral and waveform properties (Supplementary information, Fig. S5a–g).^63^ The scored results were examined and manual adjustments were made as needed. Based on the scored sleep stages, statistical analysis was performed on different vigilance states and groups.

### Histology and immunohistochemistry

To verify viral expression and optical fiber placement, mice were deeply anesthetized and immediately perfused with 0.1 M PBS, followed by 4% paraformaldehyde (PFA). After being removed from the brain, the tissues were fixed overnight in 4% PFA and then dehydrated in a 30% sucrose solution. Brain samples were frozen in an embedding medium (Cat# NEG-50, ThermoFisher Scientific, USA), sectioned into 30-μm slices using a cryostat (HM525 NX, ThermoFisher Scientific, USA), and stored at –20 °C until further processing.

Brain sections were prepared for immunostaining by permeabilization with PBST (0.3% Triton X-100 in PBS) for 30 min, blocked with 2% normal goat serum or normal donkey serum for 1 h, and then incubated with a primary antibody overnight at 4 °C. The brain sections were washed in PBS before being incubated with a secondary antibody. Finally, brain sections were washed in PBS and mounted with mounting media. The following primary or secondary antibodies were used in the current study at the indicated dilutions: chicken anti-mCherry (Cat# ab205402, Abcam, 1:2000), rabbit anti-CRE (Cat# 69050–3, Novagen, 1:3000), rabbit anti-GFAP (Cat# ab7260, Abcam, 1:500), mouse anti-NeuN (Cat# ab77487, Abcam, 1:500), rabbit anti-GABA (Cat# PA5-32241, Invitrogen, 1:2000), guinea pig anti-vGluT2 (Cat# AB2251-1, Abcam, 1:200), donkey anti-chicken Alexa Flour 488 (Cat# A78948, Invitrogen, 1:1000), goat anti-guinea pig Alexa Fluor 647 (Cat# A21450, Invitrogen, 1:1000), donkey anti-rabbit Alexa Fluor 594 (Cat# A21207, Invitrogen, 1:1000), and goat anti-mouse Alexa Fluor 647 (Cat# A21235, Invitrogen, 1:1000). The stained sections were visualized using either a confocal microscope (LSM900, Zeiss, Germany) or an epifluorescence microscope (DM6B, Leica, Germany).

To verify adenosine spreading after microinjection, 2 µL 1% fluorescein sodium (solved in ACSF) was injected into V1 using a tubing-nested 10-µL Hamilton syringe through the cannula, then after 1 h, mice were deeply anesthetized and immediately perfused with 0.1 M PBS, followed by 4% PFA. After being removed from the brain, the tissues were fixed overnight in 4% PFA and then dehydrated in a 30% sucrose solution. Brain samples were frozen in the embedding compound, sectioned into 40-μm slices using a cryostat (HM525 NX, ThermoFisher Scientific, USA), and stored at 4 °C until fluorescence scanning.

The quantification of NeuN was calculated by ImageJ. In brief, NeuN staining was visualized in Z-stack mode by confocal microscope. The mean fluorescence intensity in V1 was measured under the same signaling threshold and represented by mean gray value (signals divided by area) in ImageJ.

### Targeted UHPLC-MS/MS

The mice were randomly assigned to one of three groups: the normal light group, the 30 min post-40 Hz flickering visual stimulation group, and the 240 min post-40 Hz flickering stimulation group. After flickering visual stimulation, the mice were sacrificed rapidly and the visual cortex was promptly collected, frozen in liquid nitrogen, and stored at –80 °C until further analysis. To precipitate proteins from tissues, 200 μL of cold methanol:acetonitrile mixture (v:v = 1:1) was added, vortexed for 180 s, and the resulting supernatant was collected by centrifugation. The obtained supernatant was aliquoted into clean tubes, dried under N_2_ flow, and then re-dissolved in 100 μL of 1:1 (v:v) mixture of acetonitrile and water for subsequent UHPLC-MS/MS analysis.

A targeted assay was used to measure the levels of ATP, ADP, AMP, cAMP, IMP, Hcy, SAH, SAM, Met, inosine, and adenosine in V1 tissue. The analyses were conducted using a SHIMADZU CBM-30A Lite LC system together with an API 6500 Q-TRAP mass detector (AB SCIEX, USA), which was operated in ESI+ mode. To separate the molecules, a Kinetex C18 100A column (100 × 2.1 mm, 2.6 μm) was utilized.

Standard solutions of ATP, ADP, AMP, cAMP, IMP, Hcy, SAH, SAM, Met, inosine, and adenosine (Sigma-Aldrich, purity > 98%) were prepared at concentrations ranging from 1 to 1000 ng/mL and concentration calibration curves were established. Metabolite concentrations were determined using AB Sciex MultiQuant software (version 2.1, AB SCIEX, USA).

### HPLC analysis of total tissue adenosine

The mice were randomly divided into four groups: normal light + vehicle, normal light + dipyridamole, 40 Hz flickering + vehicle, and 40 Hz flickering + dipyridamole. After the 30-min light flickering session, the mice were sacrificed by rapid decapitation, and the V1 area was dissected and stored at –80 °C until further analysis. To precipitate proteins from V1, a mixture of precooled adenosine inhibitors (500 μL of 1 mg/mL dipyridamole and 20 μL of 20 μg/mL EHNA) and acetonitrile in a 1:19 ratio (v:v) was added, and the sample was vortexed for 0.5 min and then centrifuged at 12,000 rpm for 15 min. The supernatant (100 µL) was injected into the HPLC system for analysis. Adenosine was separated by a Polaris 5 C18-A column (Agilent, 2.1 mm × 150 mm, 5 μm) at a temperature of 25 °C, with a detection wavelength of 258 nm. Isocratic elution was performed using a mobile phase consisting of 0.1% methanol, 0.1% (phase A), and 99.8% (phase B) at a flow rate of 1 mL/min. The mobile phase B was prepared with 25 mM sodium dihydrogen phosphate and adjusted to pH 7.0 using triethylamine.

### Real-time qPCR analysis

To obtain V1 tissue, mice were first anesthetized with isoflurane. Then, V1 tissues were microscopically dissected from the brain and quickly transferred to TRIzol reagent (Invitrogen, USA). RNA quantity and quality were assessed using a NanoDrop spectrophotometer. The reverse transcription process was carried out using a PrimeScriptTM RT reagent kit (Vazyme, R323, China). The primers used for qRT-PCR included *ENT1* (5′-CAGCCTCAGGACAGGTATAAGG-3′ and 5′-GTTTGTGAAATACTTGGTTGCGG-3′), *ENT2* (5′-TCATTACCGCCATCCCGTACT-3′ and 5′-CCCAGTTGTTGAAGTTG AAAGTG-3′), *ADA* (5′-ACCCGCATTCAACAAACCCA-3′ and 5′-AGGGCGATGCCTC TCTTCT-3′), *ADK* (5′-AGAGTCGGTATTGAAAGTGGCT-3′ and 5′-CTAAAGCAA CTCACCGTCTCAT-3′), *AMPKα1* (5′-GTCAAAGCCGACCCAATGATA-3′ and 5′-CGTACACGCAAATAATAGGGGTT-3′), *AMPKα2* (5′-CAGGCCATAAAGTGGCAGTTA-3′ and 5′-AAAAGTCTGTCGGAGTGCTGA-3′), *A*_*1*_*R* (5′-GCCATCCCATTCGCCATCA-3′ and 5′-GCAATAGCCAAGAGGCTGAAGA-3′), *A*_*2A*_*R* (5′-GCCATCCCATTCGCCATCA-3′ and 5′-GCAATAGCCAAGAGGCTGAAGA-3′) and *A*_*2B*_*R* (5′-AGCTAGAGACGCAAGACGC-3′ and 5′-GTGGGGGTCTGTAATGCACT-3′). The expression levels of these genes were normalized based on the housekeeping gene *β-actin*. The SYBR Green fluorescence system was used for quantitative mRNA analysis with a StepOnePlus Real-Time PCR System (Life Technologies, USA), following the manufacturer’s instructions. Then, the mRNA expression level was determined using the ΔΔCt method, and *β-actin* was used as the calibrator gene. Target gene expression was determined as relative expression levels and each sample was analyzed at least in triplicates.

### Western blot analysis

Primary visual cortices (V1) were quickly removed from euthanized C57BL/6J mice and stored at –80 °C. Tissues were lysed by sonication in ice-cold RIPA lysis buffer (Cat# P0013B, Beyotime, China) with protease inhibitor cocktail (Cat# B14001, Bimake, USA) and phosphatase inhibitors mix (Cat# B15001, Bimake, USA), kept for 30 min at 4 °C before centrifugation at 15,000× *g* for 10 min at 4 °C. The supernatant was collected and the protein concentration was estimated using Enhanced BCA Protein Assay Kit (Cat# P0010S, Beyotime, China). Samples were diluted in 5× sodium dodecyl sulfate-polyacrylamide gel electrophoresis buffer (Cat# P0015, Beyotime, China) and boiled at 95 °C for 10 min. Next, the samples were subjected to dodecyl sulfate-polyacrylamide gel electrophoresis on a 10% gel. Anti-Phospho-AMPKα (1:1000, Cat#2535 S, Cell Signaling Technology), anti-AMPKα (1:1000, Cat# 2532S, Cell Signaling Technology), and anti-β-Actin (1:2000, Cat# 66009-1, Proteintech) antibodies were used to evaluate the relative amount of phospho-AMPKα.

### The safety and tolerability assessment of 40 Hz flickering glass on healthy controls

#### Participant recruitment

We recruited 16 healthy controls at the first affiliated hospital of Wenzhou Medical University to assess the safety and tolerance of 40 Hz light flickering on August 20th, 2021. The following were the criteria for inclusion: (1) ages from 18 to 80; (2) BMI between 19 and 25 kg/m^2^; (3) >5 years of education; (4) voluntarily signing the informed consent. The following were the criteria for exclusion: (1) drug or alcohol abuse within 12 months; (2) unable to complete the questionnaire due to visual or hearing impairment; (3) refusal to sign informed consent. This study was agreed upon by the Medical Ethics Committee of Eye Hospital of Wenzhou Medical University (NO. 2021-130-K-99-01) and abided by the Declaration of Helsinki’s principles.

#### Procedures

This was a self-controlled study. Participants were admitted into the outpatient department and were measured for blood pressure and electrocardiogram (ECG). Venous blood was also obtained as the baseline. The 40 Hz intervention was carried out in a dimmed meeting room and participants wore flickering glass stimulating at 40 Hz for 30 min in the Department of Neurology of the first affiliated hospital of Wenzhou Medical University. The 40 Hz flickering intensity was 2000 lux and 50% duty cycle (irradiance: 0.58 mW/cm^2^, CCT is 3800 K, Wenzhou Jiukuan Technology Co., Ltd., China). Participants were monitored during this 30 min-period and were forced to be awake and silent. Then these participants stopped the flicker and were reevaluated for blood pressure, ECG, and venous blood as post-flicker.

### Blood sample evaluation and adverse event record

We evaluated the vein blood for hemoglobin, white blood cell counts, glutamic-pyruvic transaminase, creatinine, and other factors to detect the potential side effects of 40 Hz flicker such as hepatic or renal insufficiency. We also recorded the adverse events and the corresponding degree as well as the relevance to flicker.

### IOP measure

IOP measurements of humans were obtained using the Optical Response Analyzer (Reichert Corp., USA) and have been described previously.^64^ In brief, both corneal-compensated and Goldman-correlated IOP measurements were collected. We used corneal-compensated IOP for this study since it is less affected by corneal thickness. The average of both eyes was used for downstream analysis.

### OCT imaging

The swept-source optical coherence tomography (SS-OCT) system (VG200S; SVision Imaging, China) contained an SS laser with a central wavelength of approximately 1050 nm and a scan rate of 200,000 A-scans per second. The system was equipped with an eye-tracking utility based on an integrated confocal scanning laser ophthalmoscope to eliminate eye-motion artifacts. The axial resolution was 5 μm, and the lateral resolution was 13 μm. The scan depth was 3 mm. SS-OCT image was performed using a previously described method.^65^ Briefly, structural OCT of the retina macular was performed with 18-radian scan lines centered on the fovea area. Each scan line, generated by 2048 A-scan, was 12 mm long and separated from the adjacent lines by 10 °. Only horizontal scan images were used to analyze the thickness of the retina and choroidal. All analysis was performed by MATLAB R2017a which referenced these studies.^66,67^

### The safety and tolerability assessment of 40 Hz flickering on mice

IOP measurements were performed using TonoLab rebound tonometry (Icare, Finland) in isoflurane-anesthetized (2.5% isoflurane and 0.8 L/min O_2_) mice as previously described.^68^ After 40 Hz light flickering (19:30–20:00), night-time IOPs were measured at the first stimulus for 30 min and last flicking at continuous for 14 days. An average of six individual readings was considered as one recording. The both eyes were used for downstream analysis.

Mice were anesthetized and pupils were dilated. 0.3% levofloxacin drop (Shenyang Sinqi Pharmaceutical Co., Ltd., China) was applied to the corneal surface. After the mouse was anesthetized, OCT scans were taken using an image-guided Spectralis OCT (Heidelberg, Germany). For the quantitative assessment of the retina reflectivity, we used the Freehand tool in ImageJ to delineate the retina thickness in OCT images and determine their mean intensity.

### Clinical study on the effect of 40 Hz flicker on children with insomnia symptoms

#### Participant recruitment

The study was approved by the Ethics Committees of the School of Optometry and Ophthalmology and Eye Hospital of Wenzhou Medical University (No. 2022-130-K-99-01) before initiation and was carried out in compliance with the World Medical Association Declaration of Helsinki. This study was conducted between February 2022 and May 2023 at the Department of Pediatrics, Second Affiliated Hospital, and Yuying Children’s Hospital of Wenzhou Medical University. Demographic data were collected from pediatricians, and any identifying information was eliminated to ensure anonymity. Data analysis was conducted by investigators blind to patients’ data and study design, and then interpreted by all authors. Potential participants and their parents were provided with detailed information regarding the purpose and procedures of the study (Chinese Clinical Trials. gov identifier: ChiCTR2300070938), and they were required to sign an informed consent form before participating (original copies are available upon request to the corresponding author). The inclusion criteria were as follows: (1) children aged 4–16 years; (2) a diagnosis of insomnia for the first time according to the International Classification of Sleep Disorders, Third Edition (ICSD-III) without any prior treatment; (3) signed informed consent forms from both patients and their parents. The study’s exclusion criteria were: (1) a history of neurological, psychiatric, or sleep disorders; (2) caffeine abuse, or the use of psychotropic medications within the past 12 months.

#### Procedures

The study followed a self-controlled design, and PSG recordings were performed for two consecutive nights on all enrolled patients. On the first day, after habituation for at least half a day, baseline sleep parameters were measured. On day 2, patients underwent a 30-min session of 40 Hz light flickering using a flickering glass device with 2000 lux (irradiance: 0.58 mW/cm^2^; CCT: 3800 K, Wenzhou Jiukuan Technology Co., Ltd.) from 8:30 to 9:00 PM, and sleep parameters were recorded for the entire night. Demographic data, such as the participant’s name, gender, weight (kg), height (cm), and calculated BMI, were collected. Before the study began, all patients underwent extensive history recording, including presenting complaints and history of presenting complaints, and were given half a day to acclimate to the ward environment. Whole-night sleep recordings were obtained using PSG equipment (Embla s4500, USA). Airflow was evaluated using a nasal pressure and/or oronasal thermistor sensor, while heart rate and arterial blood oxygen saturation levels were monitored using a pulse oximeter (Nonin Medical Inc., USA) during night-time sleep. These parameters were obtained simultaneously and continuously throughout the night. Sleep stages were classified based on the 2012 Academy of Sleep Medicine manual guidelines.^60^

#### Outcomes

The primary objective was to evaluate the effects of 40 Hz flickering on sleep onset by measuring SOL (minutes from light off to sleep onset) with PSG and comparing it to baseline. The secondary outcomes were to assess improvement in sleep maintenance parameters such as TST (sleep period time minus the duration of intra-sleep awakenings), SE (total sleep time/total time in bed), WASO (minutes of wake after sleep onset), and AF (the number of nightly awakenings) compared to baseline. In addition to primary and secondary endpoints, this study also evaluated other sleep parameters including REM sleep onset latency (REM SOL, the time in minutes between sleep onset and the first epoch of REM sleep), percentage of light sleep (N1 + N2), and percentage of deep sleep (N3).

### Statistical analysis

The analysts were blinded to interventions during the data analysis. GraphPad Prism 9.0 software (GraphPad Software) was utilized for statistical analysis. Data normality was checked using the Kolmogorov–Smirnov test. If the data were normally distributed, the mean ± SEM was used to express it, and a paired Student’s *t*-test was applied. Data that did not meet a normal distribution were reported as median (lower quartiles-upper quartiles) and analyzed with the Wilcoxon matched-pairs signed rank test. *P* < 0.05 was considered statistically significant. For sleep time-course analysis, two-way ANOVA was performed and significance at each time point was evaluated by Student’s *t*-test.

### Supplementary information

Supplementary Figure 1

Supplementary Figure 2

Supplementary Figure 3

Supplementary Figure 4

Supplementary Figure 5

Supplementary Figure 6

Supplementary Figure 7

Supplementary Figure 8

Supplementary Tables S1 and S2

## Data Availability

The data that support the findings of this study are available from the corresponding author, upon reasonable request.
