# Supplementary material for: 40 Hz light flickering promotes sleep through cortical adenosine signaling
Source: Cell Res. 2024 Feb 8;34(3):214–31. doi: 10.1038/s41422-023-00920-1 (PMC10907382; doi:10.1038/s41422-023-00920-1)
Supplement: Supplementary file 1 — Supplementary Figure 1 [file 41422_2023_920_MOESM1_ESM.pdf]

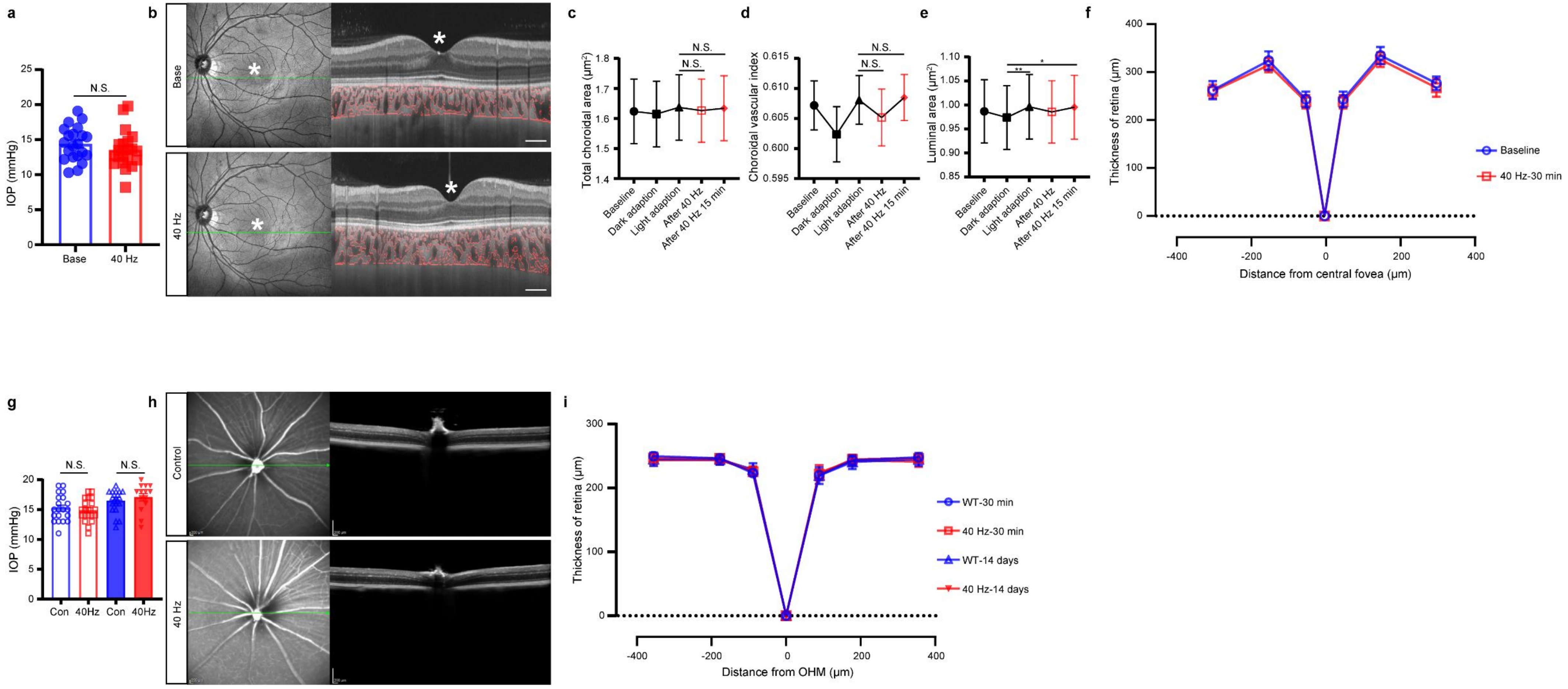

Fig. S1 40 Hz light flicker for 30 min did not affect choroid hemodynamics and optical pressure in human (a-e) and mice (f-h). a There was no significant difference in intraocular pressure of human before and after 40 Hz light flickering for 30 min. **b** The images of the human choroid by swept-source optical coherence tomography (SS-OCT). **c-e** Quantification of the choroidal vascular index, total choroidal area and luminal area of the adult volunteers. After dark adaptation, light adaptation increased choroid vascular index, but 40 Hz flickering did not produce significant effect on choroid hemodynamics (n= 10/group with 20 eyes/ group). **f** Quantification of the thickness of the retina (not including choroid) from 14 volunteers. There was no significant difference in the thickness of the macular zone by SS-OCT between light adaption and 40 Hz stimulus for 30 min (n= 14/group). **g** No significant difference was observed in intraocular pressure of mice between control group and 40 Hz light flicker after 30min and 14 days. **h** The images of the mice fundus and retina by optical coherence tomography. **i** Quantification of the thickness of the retina (not including choroid) from mice. There was no significant difference in the thickness of the mice retina by optical coherence tomography (n= 10/group).
