# Supplementary material for: 40 Hz light flickering promotes sleep through cortical adenosine signaling
Source: Cell Res. 2024 Feb 8;34(3):214–31. doi: 10.1038/s41422-023-00920-1 (PMC10907382; doi:10.1038/s41422-023-00920-1)
Supplement: Supplementary file 2 — Supplementary Figure 2 [file 41422_2023_920_MOESM2_ESM.pdf]

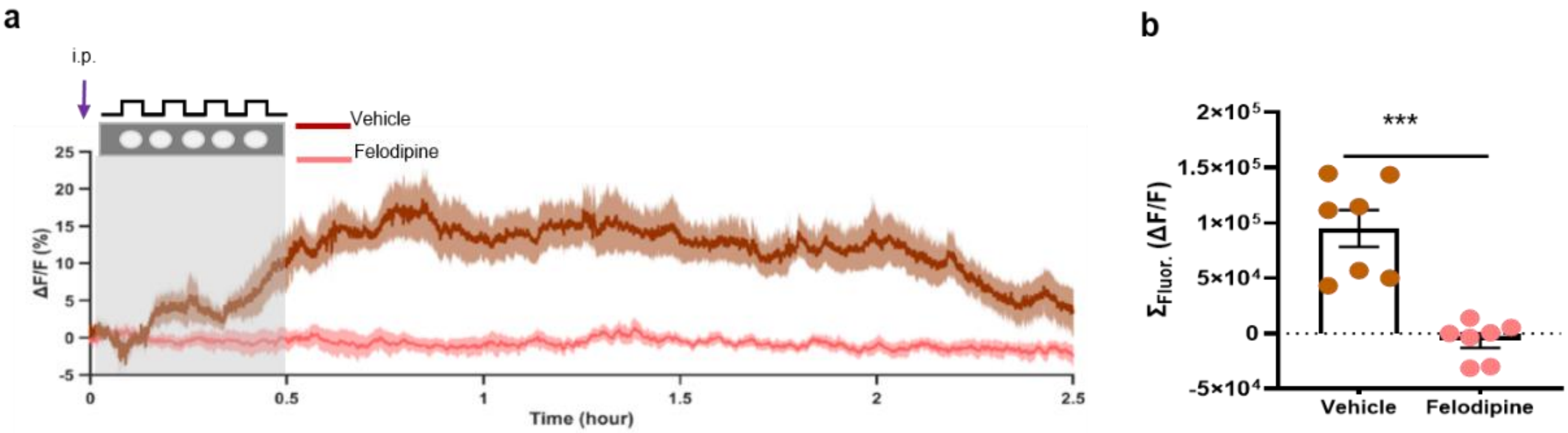

Fig. S2 40 Hz flashing -dependent Ado release is blocked by inhibitors of L-type voltage-gated calcium channels (VGCCs). a Pretreatment with an L-type VGCC inhibitor felodipine (10 mg/kg, i.p.) abolished the increased extracellular adenosine levels induced by 40 Hz flashing (n = 7/group). **b** Quantification of L-type VGCC inhibitor felodipine-abolished adenosine signaling response to 40 Hz flickering in (**a**). The data are presented as mean ± SEM, ***$P < 0.001$, Student's t-test; felodipine-treated group vs. vehicle group.
