# Supplementary material for: 40 Hz light flickering promotes sleep through cortical adenosine signaling
Source: Cell Res. 2024 Feb 8;34(3):214–31. doi: 10.1038/s41422-023-00920-1 (PMC10907382; doi:10.1038/s41422-023-00920-1)
Supplement: Supplementary file 3 — Supplementary Figure 3 [file 41422_2023_920_MOESM3_ESM.pdf]

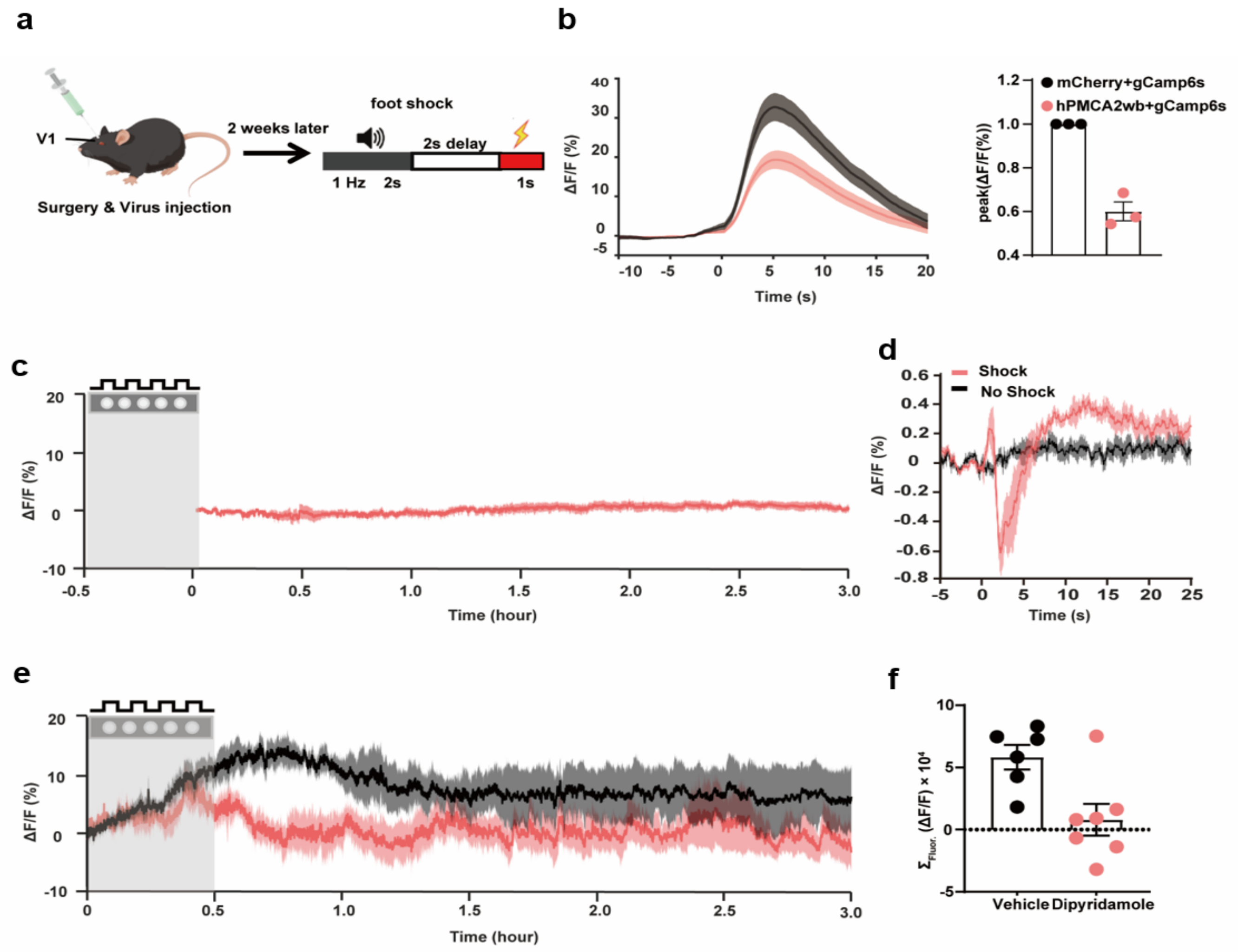

Fig. S3 The sustained increase in extracellular adenosine was dependent on ENT activity not ATP release. a Behavioral protocol for reward and foot shock conditioning tasks after expression of hPMCA2w/b in V1 two week. **b** Expression of hPMCA2w/b in V1 partially reduced astrocytic activity, as indicated by calcium signaling using gCaMP detector during foot shock conditioning tasks (n = 3/group). **c & d** Measurement of extracellular ATP levels after 40 Hz flickering (left figure) and after electric shock in mice expressing $GRAB_{ATP}$ (right figure) (n = 6/group). The data are presented as mean ± SEM, $**P < 0.01$, $*P < 0.05$, Student's t-test; dipyridamole-treated group vs. vehicle group. **e & f** Administering dipyridamole immediately after light flickering also eliminated 40 Hz flickering-induced subsequent extracellular adenosine generation after light flashing cessation (n = 6/group). The data are presented as mean ± SEM, $**P < 0.01$, $*P < 0.05$, Student's t-test; dipyridamole-treated group vs. vehicle group.
