# Supplementary material for: 40 Hz light flickering promotes sleep through cortical adenosine signaling
Source: Cell Res. 2024 Feb 8;34(3):214–31. doi: 10.1038/s41422-023-00920-1 (PMC10907382; doi:10.1038/s41422-023-00920-1)
Supplement: Supplementary file 4 — Supplementary Figure 4 [file 41422_2023_920_MOESM4_ESM.pdf]

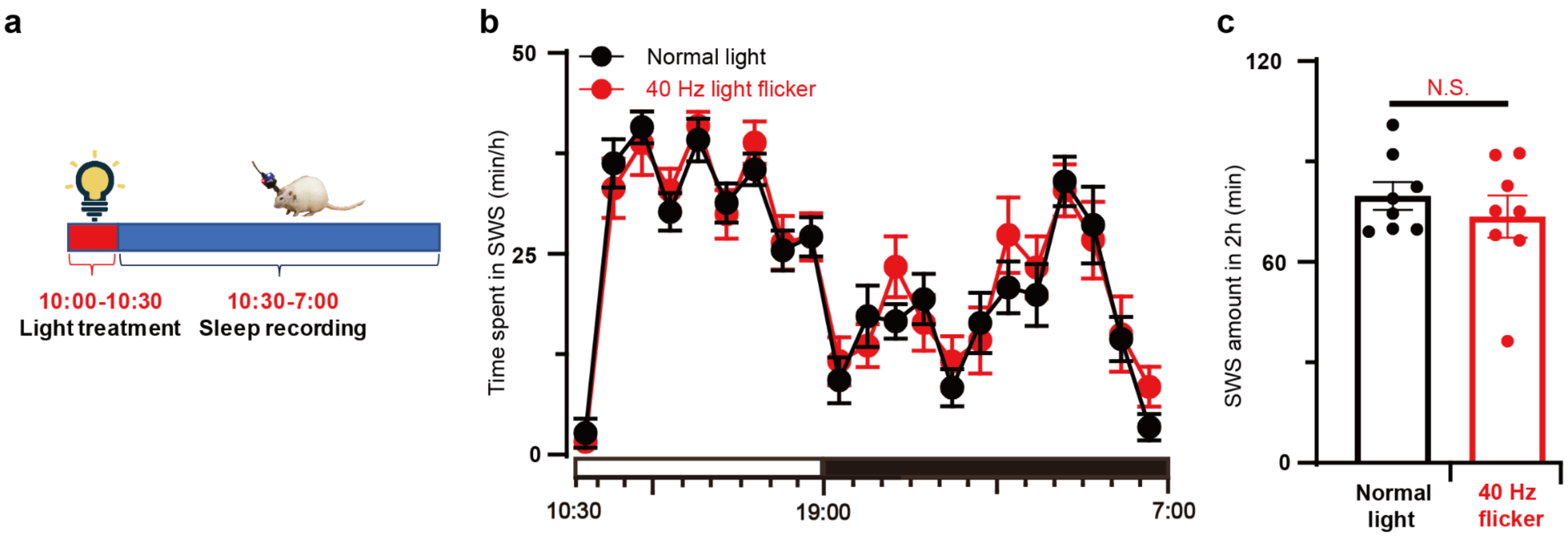

Fig. S4 Daytime 40 Hz light flicker treatment cannot increase SWS amount in WT mice. a Experimental design for daytime light treatment and sleep recordings. **b** Time-course of SWS in mice after normal light and 40 Hz light flicker treatments, respectively. (light treatment: 10:00 am-10 :30 am). **c** Amount of NREM and REM sleep in first 2 hours after normal light and 40 Hz light flicker treatments in mice, respectively. *p<0.05; 40 Hz VS normal light, significance was assessed by Student's t-test.
