# Supplementary material for: 40 Hz light flickering promotes sleep through cortical adenosine signaling
Source: Cell Res. 2024 Feb 8;34(3):214–31. doi: 10.1038/s41422-023-00920-1 (PMC10907382; doi:10.1038/s41422-023-00920-1)
Supplement: Supplementary file 5 — Supplementary Figure 5 [file 41422_2023_920_MOESM5_ESM.pdf]

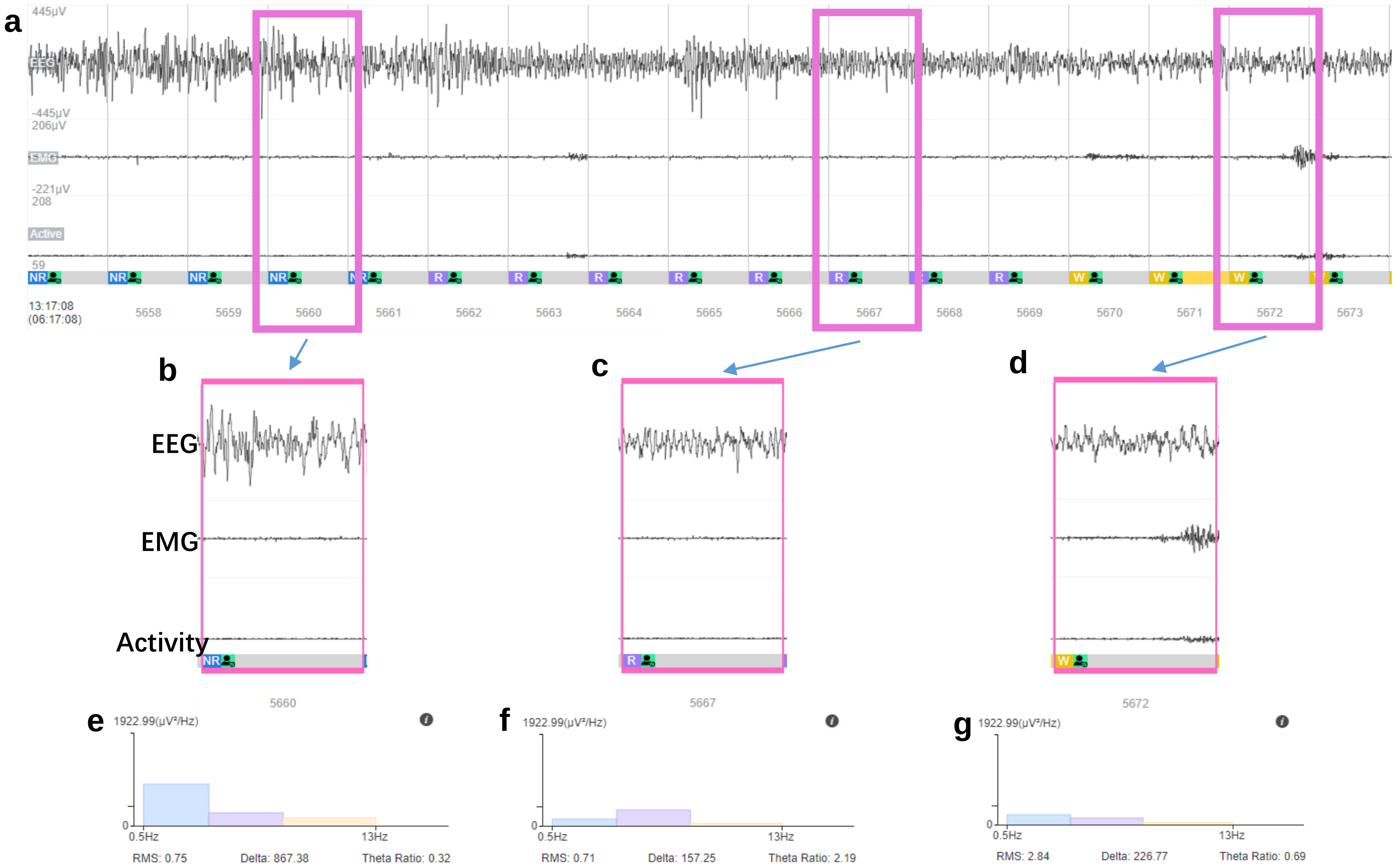

Fig. S5 Real operation window of vigilance scoring. a paradigm of EEG/EMG/Activity raw data in a 80-s recording period. **b-d** Typical examples of SWS (labeled as NR), REM sleep (labeled as R) and wakefulness (labeled as W) in a 4-s epoch counting unit, respectively. **e-g** Typical examples of FFT of SWS (matching with **b**), REM sleep (matching with **c**) and wakefulness (matching with **d**) in a 4-s epoch counting unit, respectively.
