# Supplementary material for: 40 Hz light flickering promotes sleep through cortical adenosine signaling
Source: Cell Res. 2024 Feb 8;34(3):214–31. doi: 10.1038/s41422-023-00920-1 (PMC10907382; doi:10.1038/s41422-023-00920-1)
Supplement: Supplementary file 6 — Supplementary Figure 6 [file 41422_2023_920_MOESM6_ESM.pdf]

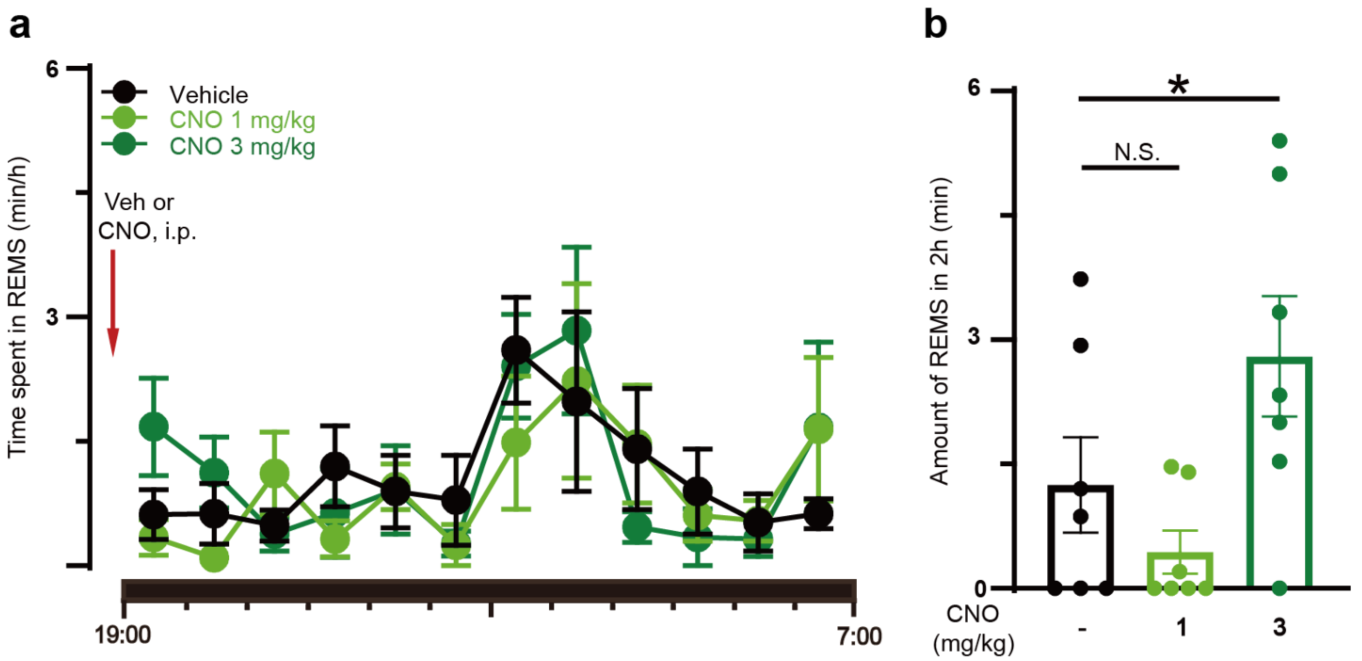


**Fig. S6 Chemogenetic inhibition of V1 neurons by hM4Di recaptures the increase of REMS in mice. a** Time-course of REMS after vehicle and CNO (1 mg/kg and 3 mg/kg) injections. **b** CNO at 3 mg/kg significantly increased REMS in mice. *$p<0.05$ vs vehicle, significance was assessed by one-way ANOVA.
