# Supplementary material for: 40 Hz light flickering promotes sleep through cortical adenosine signaling
Source: Cell Res. 2024 Feb 8;34(3):214–31. doi: 10.1038/s41422-023-00920-1 (PMC10907382; doi:10.1038/s41422-023-00920-1)
Supplement: Supplementary file 7 — Supplementary Figure 7 [file 41422_2023_920_MOESM7_ESM.pdf]

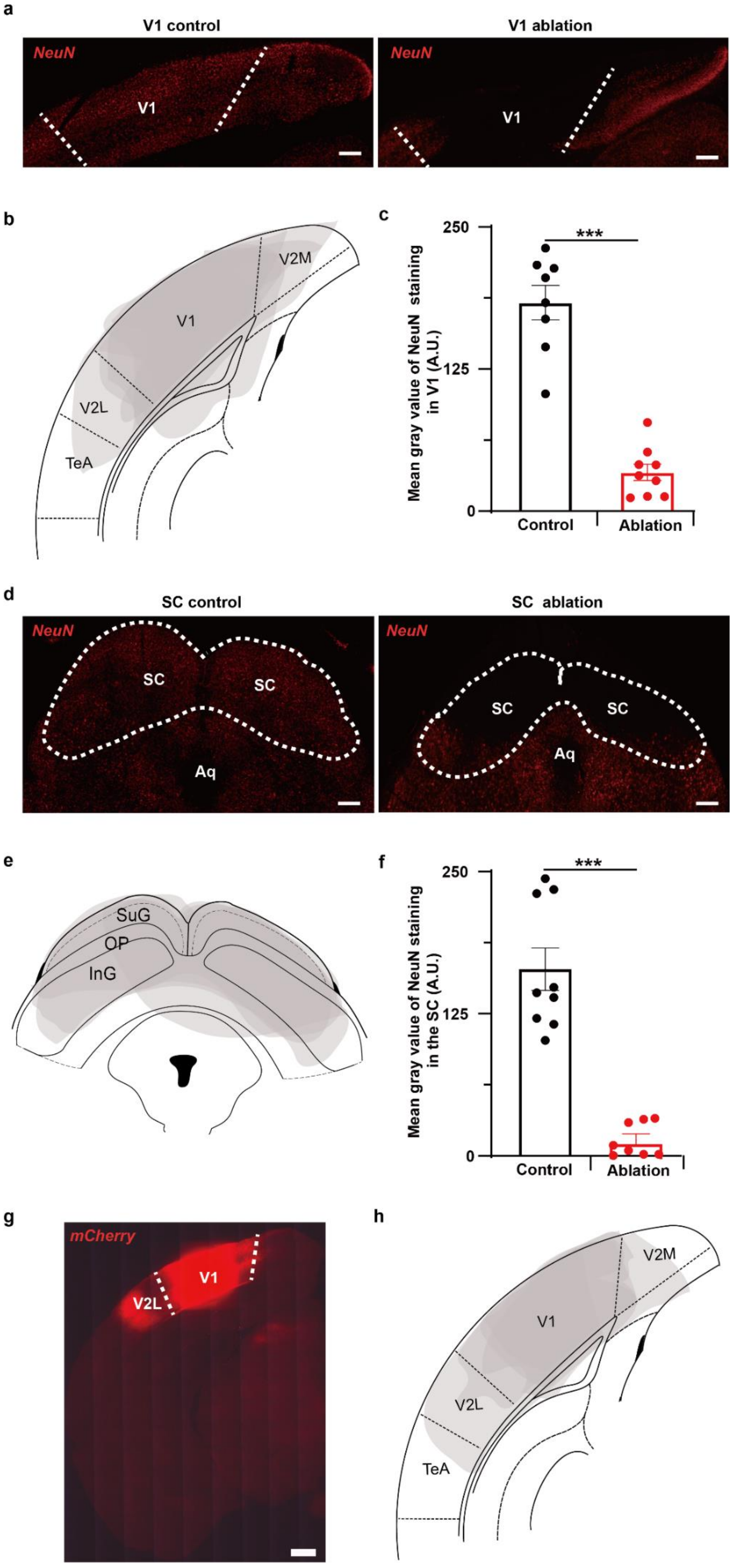

Fig. S7 Ablation of V1 or SC neurons by AAV-hSyn-taCaps3 and expression of AAV-hSyn-hM4Di in V1. **a** Fluorescence staining of NeuN in V1 ablation and control mice, respectively. Bar= 500 μm. **b** Superimposed gray shadow indicates ablation area of AAV-hSyn-taCaps3 in V1. **c** Statistical analysis of mean fluorescence intensity (reflected by mean gray value) of NeuN signals in V1 in control and ablated mice. ***p<0.001 vs control, assessed by Students' t-test. **d** Fluorescence staining of NeuN in SC ablation and control mice, respectively. Bar = 500 μm. **e** Superimposed gray shadow indicates ablation area of AAV-hSyn-taCaps3 in the SC. **f** Statistical analysis of mean fluorescence intensity (reflected by mean gray value) of NeuN signals in the SC in control and ablated mice. ***p<0.001 vs control, assessed by Students' t-test. **g** Spontaneous fluorescence of mCherry indicates AAV-hSyn-hM4Di-mCherry expression in V1. Bar = 500 μm. **h** Superimposed gray shadow indicates mCherry expression area of AAV-hSyn-hM4Di-mCherry in V1. V2M, medial secondary visual cortex; V2L, lateral secondary visual cortex; TeA, temporal association cortex; SuG, superficial gray layer of the superior colliculus; OP, optic nerve layer of the superior colliculus; InG, intermediate gray layer of the superiorcolliculus.
