# Supplementary material for: 40 Hz light flickering promotes sleep through cortical adenosine signaling
Source: Cell Res. 2024 Feb 8;34(3):214–31. doi: 10.1038/s41422-023-00920-1 (PMC10907382; doi:10.1038/s41422-023-00920-1)
Supplement: Supplementary file 8 — Supplementary Figure 8 [file 41422_2023_920_MOESM8_ESM.pdf]

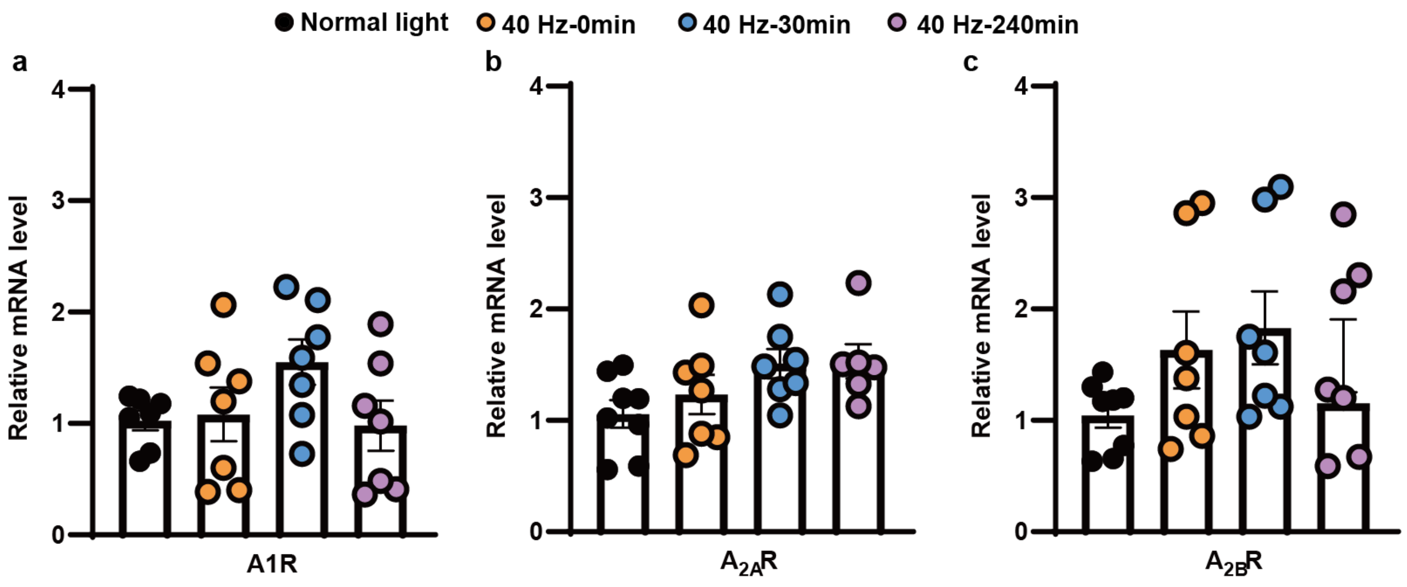

Fig. S8 The mRNA expression of A1R, $A_{2A}R$ and $A_{2B}R$ was not affected by 40 Hz light flicking. a-c The effect of 40 Hz light flicking on the mRNA expression of A1R (**a**), $A_{2A}R$ (**b**) and $A_{2B}R$ (**c**), n=7.Yellow: sampling was performed immediately after 40 Hz flicker; Blue: 30 min after 40 Hz flicker, sampling was performed; Purple: 240 min after 40 Hz flicker, sampling was performed.
