# Supplementary material for: 40 Hz light flickering promotes sleep through cortical adenosine signaling
Source: Cell Res. 2024 Feb 8;34(3):214–31. doi: 10.1038/s41422-023-00920-1 (PMC10907382; doi:10.1038/s41422-023-00920-1)
Supplement: Supplementary file 9 — Supplementary Tables S1 and S2 [file 41422_2023_920_MOESM9_ESM.docx]

| **Table S1 Safety evaluation of the 40 Hz light flickering for 30 min in 30**  **healthy volunteers** | | | |
| --- | --- | --- | --- |
|  | Pre (n=16) | Post (n=16) | P-value |
| Heart rate (bpm) | 71.13±9.86 | 69.94±11.86 | 0.534 |
| Systolic pressure (mmHg) | 106.94±8.68 | 106.06±8.85 | 0.614 |
| Diastolic pressure (mmHg) | 67.81±6.56 | 67.25±6.99 | 0.736 |
| Hyperlipidemia, n (%) | 9 (56.25) | 9 (56.25) | 1 |
| Hyperuricemia, n (%) | 4 (25.00) | 5 (31.25%) | 1 |
| Pathoglycemia, n (%) | 0 (0) | 0 (0) | 1 |
| Hepatic insufficiency, n (%) | 0 (0) | 0 (0) | 1 |
| Renal dysfunction, n (%) | 0 (0) | 0 (0) | 1 |
| Anemia, n (%) | 1 (6.25) | 1 (6.25) | 1 |
| Arrhythmia, n (%) | 0 (0) | 0 (0) | 1 |
| Epilepsy, n (%) | 0 (0) | 0 (0) | 1 |
| Note: Data were analyzed using paired t-test or chi-square test. Data of heart rate, systolic pressure and diastolic pressure are presented as mean ± SD | | | |

| **Table S2 Summary of adverse events after 40 Hz light flickering for 30 min in 30 healthy volunteers** | | | | |
| --- | --- | --- | --- | --- |
| Adverse Event | Numbers | Severity | Subjects affects  (No.) | Related to treatment |
| Diarrhea | 1 | mild | 15 | No |
| Eye dryness | 2 | mild | 2,3 | Possible |
| Dizziness | 2 | mild | 8 | Possible |
| Lacrimation | 1 | mild | 11 | Possible |
| Sinus bradycardia | 1 | mild | 15 | No |
| T-wave change | 1 | mild | 8 | No |
| Early ventricular repolarization | 1 | mild | 14 | No |
